# Exploring the past, present, and future of the mindfulness field: A multitechnique bibliometric review

**DOI:** 10.3389/fpsyg.2022.792599

**Published:** 2022-07-26

**Authors:** Aldijana Bunjak, Matej Černe, Emilie Lara Schölly

**Affiliations:** ^1^Institute for Leadership and Human Resource Management, University of St. Gallen, St. Gallen, Switzerland; ^2^School of Economics and Business, University of Ljubljana, Ljubljana, Slovenia

**Keywords:** mindfulness, bibliometric review, document co-citation analysis, co-word analysis, bibliographic coupling

## Abstract

This paper provides an overview of the mindfulness literature up until the end of 2020 by (a) uncovering its underlying intellectual structure, (b) identifying the most influential and popular themes, and (c) presenting new directions for future research on mindfulness. To this end, a systematic quantitative review based on bibliometric methods was conducted, which is perhaps less prone to researcher bias and can complement existing meta-analyses and qualitative (narrative) structured reviews as an objective approach. Three bibliometric techniques—document co-citation analysis, co-word (co-occurrence and content) analysis, and bibliographic coupling—were applied to explore the past, present, and future of mindfulness research. The co-citation analysis showed that measurement, mechanisms, mindfulness-based interventions, and examinations of the efficacy of mindfulness interventions are among the key theoretical knowledge bases from which the field of mindfulness is derived. The content analysis demonstrated the beneficial effects of mindfulness meditation for physical and mental health conditions. The bibliographic coupling revealed novel directions in cognitive behavioral therapy, emotion regulation, the application of mindfulness practice to children and adolescents, mindfulness at work, and the role of mindfulness in positive psychology. The large sample of articles that was analyzed allowed us to provide a broader and more objective overview than possible with other forms of literature reviews. The combination of the three bibliometric techniques granted deeper insights into the complex multidisciplinary field of mindfulness, along with specific suggestions for future research.

Mindfulness is defined as the awareness that arises through the practice of paying attention in the present moment (Kabat-Zinn, 2003). Mindfulness-based interventions have been used in the last decades, mostly in the fields of psychiatry and clinical psychology, to treat a wide variety of mental disorders and in clinical contexts to help alleviate chronic pain. In recent years, practicing mindfulness in the form of meditation has also become increasingly popular among non-clinical populations who are looking to reduce stress and improve their wellbeing (Chiesa and Serretti, 2009). During mindfulness practice, the individual directs their focus to internal (e.g., their thoughts, feelings, and bodily sensations) and external (e.g., visual events and sounds) experiences that are occurring, without automatically reacting to or judging them (Segal et al., 2002). In addition to reducing stress and improving psychological wellbeing, regular mindfulness practice has been shown to lead to positive outcomes such as increased life satisfaction, better sleep, and higher self-awareness (Kabat-Zinn, 2003; Baer et al., 2004; Fredrickson et al., 2008). Mindfulness meditation also reduces anxiety and depression symptoms significantly (Teasdale et al., 2000; Hofmann et al., 2010; de Jong et al., 2016).

To date, published research has focused largely on measuring mindfulness, developing assessment scales (Brown and Ryan, 2003; Baer et al., 2004, 2006), and evaluating the effectiveness of mindfulness-based interventions, such as the mindfulness-based stress reduction (MBSR) program (Kabat-Zinn et al., 1992; Grossman et al., 2004) and mindfulness-based cognitive therapy (MBCT; Teasdale et al., 2000). Furthermore, researchers have investigated the mechanisms of mindfulness to explain the underlying processes that link mindfulness practice to the resulting positive changes (Shapiro et al., 2006; Hoelzel et al., 2011).

Several qualitative and meta-analytic reviews that provide an overview over the mindfulness field already exist, but are mostly partial and focused on a specific subfield of mindfulness research. For example, reviews have been published on mindfulness-based interventions performed on clinical and non-clinical populations (Mason and Hargreaves, 2001; Grossman et al., 2004; Chiesa and Serretti, 2009; Hofmann et al., 2010; Khoury et al., 2013), mindfulness-measurement scales (Goodman et al., 2017), mindfulness for children and adolescents (Carsley et al., 2018), and mindfulness in organizations (Sutcliffe et al., 2016).

Recently, bibliometric analysis has become popular among researchers due to the review comprehensiveness and objectivity it enables (Zupic and Cater, 2015; Donthu et al., 2021). However, very few bibliometric reviews of mindfulness research have been conducted to date. For example, a bibliometric analysis conducted by Valerio (2016) (on research published up to 2014) showed that the conceptualization of mindfulness is evolving quickly across disciplines (e.g., education, nursing, and economics), confirming the rapid growth and importance of mindfulness in fields beyond psychology and medicine. Similarly, Chiesa et al. (2017) conducted a citation analysis of mindfulness literature (on research published up to 2016) and classified the papers by focus (e.g., clinical/non-clinical effects and type of intervention), indicating potential topics about which researchers could make future contributions. Kee et al. (2019) applied a scoping review (on research published up to 2017), indicating the range of the topics researched in the field of mindfulness based on topic modeling—that is, by combining fields based on titles and abstracts. Ma et al. (2021) have taken into account only one aspect of mindfulness, i.e., describing overarching trends in the publications of randomized controlled trials (RCTs) in their bibliometric analysis over 2000–2019. Further, Wang et al. (2021) in their bibliometric analysis use keywords referring to mindfulness and meditation. However, mindfulness and mediation may not necessarily be the synonyms (e.g., Phelan, 2010; Hanley et al., 2015). These studies merely focus on the influences of specific studies and topics without explaining the evolution of the field's development or highlighting how different but thematically similar documents cluster together in networks and thus visually portraying the field.

Recent bibliometric analysis by Baminiwatta and Solangaarachchi (2021) is focused on publications, research areas, journals authors, collaboration among authors, organizations, and countries in mindfulness research. Therefore, our work is corroborating Baminiwatta and Solangaarachchi's (2021) co-citation and co-word analysis by providing an extensive overview of publications in relevant research clusters, and advancing it by presenting the development of the field over time using the invisible colleges framework and providing a more objective take on the future trends based on bibliographic coupling. Finally, paper by Daniel et al. (2022) focused on the very definition and constituents of the mindfulness construct. This theory-driven review is highly profiled in specific topics of mindfulness (e.g., mindfulness processes, dimensions and development) and based on articles published between 2012 and 2020. Similarly, as with a previous review, we advance the existing study by including co-word (co-occurrence and content) analysis and the interpretation of the field's development that could additionally inform mindfulness research. Therefore, mindfulness research could benefit from a comprehensive review that provides a coherent, holistic, all-inclusive, and multidisciplinary overview by applying an empirically sound methodological approach.

We conducted a quantitative systematic review based on bibliometrics, covering the period until the end of 2020, to complement these studies and contribute a wider perspective, using a combination of three techniques: document co-citation analysis, co-word analysis, and bibliographic coupling (Porter et al., 2002). We are complementing the existing mindfulness literature reviews by applying some of the most advanced bibliometric techniques to tap into the historical development of mindfulness research (past), reveal the most influential topics at present, and indicate future directions in the field.

In essence, bibliometric analysis is a quantitative method of using bibliographic data—that is, the reference lists of documents—to describe, evaluate, and monitor published research in a chosen scientific field (Zupic and Cater, 2015). Bibliometric methods have the power to discern an article's importance in relation to other articles in the field and show how the different articles cluster together in a network. Thus, connections between publications are made visible, and certain patterns or trends that remain hidden to meta-analyses or structured reviews can be uncovered. Further advantages of bibliometric methods include the rigor and the level of objectivity they introduce into evaluations of scientific literature (Zupic and Cater, 2015). Researcher bias is typically not as present as it is in other types of review studies. Examples of such biases include an exaggerated focus on one specific author and frequent self-citation (Wallin, 2005). Finally, and most importantly, a much larger sample size of documents can be evaluated when applying bibliometric methods (Batistič et al., 2017). Such an analysis can easily be conducted with several 100 articles; it would be extremely cumbersome to process the same number of documents in a meta-analysis.

The attempted contribution of this paper is two-fold. On one hand, we aim to contribute to the existing mindfulness literature by providing a comprehensive (aerial) and objective review of it. On the other hand, this bibliometric review reveals the documents with the most impact on the field, their main topics of investigation, and current research trends and proposes promising directions for future research. More specifically, this paper looks for answers to three research questions: (a) What is the underlying intellectual structure of the mindfulness literature, and how has it developed over time (addressed by co-citation analysis, in combination with temporal analysis, by applying the invisible colleges framework)? (b) What are the central topics in the research field (tackled by co-word/co-occurrence analysis)? (c) What is the current state-of-the-art, and which future directions for mindfulness research are the most relevant (answered by bibliographic coupling)?

First, we provide the theoretical background of the mindfulness construct, including definitions, explanations of the underlying concept, mindfulness practices, and a range of interventions. Second, we selected and analyzed data for each of the three bibliometric techniques and present the results here. Third, we summarize the results in a general discussion and then lay out the recommendations for future research in the field of mindfulness and the potential limitations stemming from our use of bibliometric techniques.

## Theoretical background

### Origins and early research

The roots of mindfulness can be found in Eastern spiritual practices, which encourage the regular cultivation of mindfulness (Brown and Ryan, 2003). In the 1960s and 1970s, Western researchers started to translate mindfulness practices from Buddhist traditions, with the objective of making mindfulness available to a larger society (Baer, 2003; Kabat-Zinn, 2003; Shonin et al., 2013). In 1979, Kabat-Zinn achieved a breakthrough with the invention of the mindfulness-based stress reduction (MBSR) program, and soon after, in 1982, he published the first article to demonstrate the efficacy of MBSR (Kabat-Zinn, 2003). Researchers in the early 2000s concentrated on establishing valid and reliable measurement scales for mindfulness. Since then, studies have confirmed the salutary effects of mindfulness, and mindfulness research has kept growing exponentially, investigating various facets of mindfulness and its application in a wide variety of contexts.

### Definitions and measurements

Researchers have not yet reached a consensus on a single, clear definition of mindfulness (Bishop et al., 2004). Brown and Ryan (2003) provided perhaps one of the one of the most widely accepted and used definitions of mindfulness, calling it “the state of being aware and attentive to what is happening in the present” (p. 822). Different authors have conceptualized mindfulness in different manners and have developed and validated measurement scales accordingly (Levinson et al., 2014). The qualitative review of Glomb et al. (2011) offers an overview of frequently used mindfulness definitions.

A frequently debated question in the mindfulness literature is whether mindfulness is a trait or a state. A trait refers to an inherent characteristic, whereas a state describes a momentary mode of being that lasts only for a limited amount of time (Brown and Ryan, 2003). According to Baer et al. (2006), the two are not mutually exclusive, and mindfulness can be considered a concept with both trait-like and state-like qualities. In accordance with this, Brown and Ryan (2003) found that mindfulness exhibits both inter- and intrapersonal variations. This means that some people are just inherently higher in mindfulness than others, but simultaneously, a person does not display the same level of mindfulness at all times. Mindfulness also commonly has been looked at as a set of skills that can be developed with regular practice (Baer et al., 2006). Thus, although some people inherently have a higher capacity for mindfulness than their peers do, one can become more mindful with training.

Similarly, different mindfulness measurement scales have been developed over the years. Some of the mindfulness measurement scales that have been developed and employed in psychological research over the last decade include the Freiburg Mindfulness Inventory (FMI; Walach et al., 2006), the Mindful Attention Awareness Scale (MAAS; Brown and Ryan, 2003), the Kentucky Inventory of Mindfulness Skills (KIMS; Baer et al., 2004), the Five Facet Mindfulness Questionnaire (FFMQ; Baer et al., 2006), and the Toronto Mindfulness Scale (TMS; Lau et al., 2006). The FFMQ is thought to provide the most comprehensive coverage of the different aspects of mindfulness, especially for assessing levels of mindfulness among the general population (Bergomi et al., 2013). A comprehensive review by Sauer et al. (2013) summarizes the different mindfulness measurements.

### Mindfulness-based interventions and practices

Regarding mindfulness-based interventions (MBIs), a distinction must be made between interventions that have mindfulness at the center—which is the case for the MBSR MBCT programs—and interventions that incorporate mindfulness training but also comprise behavior-change strategies (Baer, 2003). Different interventions may be applied, depending on the patient's situation or condition. For instance, a meta-analysis by Eberth and Sedlmeier (2012) argued that MBSR may have the most powerful effect on attaining higher psychological wellbeing, whereas pure mindfulness meditation showed the greatest effects on variables associated with the concept of mindfulness.

Mindfulness is often practiced through meditation exercises. Kabat-Zinn (1982) defined meditation as “the intentional self-regulation of attention from moment to moment” (p. 431). Mindfulness meditation must be differentiated from transcendental meditation and other types of concentration-based approaches (Kabat-Zinn, 1982). However, mindfulness practice is not limited to meditation. One can also practice being attentive and aware in the present moment during everyday activities such as eating, walking, standing, or even washing the dishes (Hanley et al., 2015).

## Bibliometric methods and techniques

Bibliometric analysis is not necessarily a new method (Kessler, 1963), but it has gained scholarly interest in the recent past due to easily accessible online databases, which contain nearly all documents that have ever been published. This development has also been supported by new and improved bibliometric software (e.g., VOSviewer and BibExcel), which facilitate the data structuring and analysis processes significantly.

Bibliometric techniques are instruments to evaluate and analyze scientific literature and are considered a form of science mapping, which is defined as “a combination of classification and visualization” that “aims to reveal the structure and dynamics of scientific fields” (Zupic and Cater, 2015, pp. 429, 431). The main objective of bibliometric methods is to uncover the relationships between publications. These relationships are based on the coupling of items in bibliographic records, with the coupling strength measured by the number of connections between the items (Wallin, 2005). The essential idea is that we can speculate about topical similarities based on citations and collaborations in the scientific literature (Zupic and Cater, 2015). It is important to differentiate between primary and secondary documents. Primary documents form the basis of bibliometric reviews. They are the publications yielded by a keyword search in a database. Secondary documents are the documents cited by the primary documents and thus do not necessarily appear in the keyword search results (Vogel et al., 2020).

### Document co-citation

Document co-citation refers to the frequency at which two documents, authors, or journals are cited together (Small, 1973). Simply put, if two cited articles (secondary documents) appear in the same reference list of a citing article (primary document), they are co-cited (Batistič et al., 2017). A fundamental assumption of co-citation states that the more often two documents are cited together, the more likely their content and key concepts are to be related (Wallin, 2005; Zupic and Cater, 2015). The more frequently a pair of co-cited documents appears in a data set, the stronger their connection, or link strength, is (Batistič and Kaše, 2015).

### Co-word (co-occurrence)

Co-word analysis, also termed co-occurrence, originally introduced by Callon et al. (1983), is used to study a document's content. In fact, it is the only bibliometric method that uses the actual content of documents to construct a similarity measure. It involves using the important words or keywords in the documents, which Web of Science extracts from the abstract and title, to establish relationships and thus provides an overview of a field's conceptual structure (Zupic and Cater, 2015). Consequently, co-occurring words are a good indicator that documents are related in their key topics. The rule states that the higher the number of publications in which two keywords appear, the stronger the connection between the two concepts that those keywords describe. This is indicated by how closely keywords are located near one another on a map.

### Bibliographic coupling

Bibliographic coupling measures the similarity between two documents by using the number of references they share (Kessler, 1963). Consequently, the more similar the items contained by two coupled reference sections, the stronger their connection. As stated previously, the difference between bibliographic coupling and document co-citation is that the former focuses more on the citing document (primary document) rather than on the cited documents (secondary documents). Thus, bibliographic coupling is a useful method for analyzing the research front or smaller subfields that are not necessarily cited often enough to establish relevant connections in co-citation analysis. Zupic and Cater (2015) stated that this is especially the case for research areas that have recently experienced exponential growth, as the mindfulness field has. Bibliographic coupling is also better suited than co-citation is for detecting current trends and future directions in a research area because citing documents, by definition, are more recent than the publications they cite.

## Methods

To obtain answers to the research questions stated in the introduction, we employed three bibliometric techniques: (a) document co-citation, to investigate relationships between the authors of the most impactful publications and uncover the intellectual structure of the mindfulness field; (b) co-word analysis, to identify key clusters of content and their relations; and (c) bibliographic coupling, to distill current hot topics and potential directions for future research.

We were also interested in the field's development across three time periods—up to 2000, 2001–2010, and 2011–2020. We interpreted changes in the field's development over time by building on the conceptual framework of “invisible colleges” (cf. de Solla Price, 1965; Vogel, 2012), which we used to explore scientific communication between scholars to elucidate the dynamic change across the three time periods of the field's evolution. This framework is applied to interpret the academic communication between researchers to elucidate the past of the mindfulness field. Exploring this allows us to present the changing perspective of emerging and shifting colleges of literature that mindfulness studies have cited in a specific time period.

Vogel (2012) proposed seven patterns by which invisible colleges evolve: college appearance, transformation, drift, differentiation, fusion, implosion, and revival. College appearance is the emergence of a new college without predecessor in the same field, even though its foundations may be long-standing. College transformation is the gradual or sudden change of an existing college, which may result in the formation of a new college. To some extent, this evolutionary pattern is universal because all colleges change over time. This is true even for colleges that show a high degree of continuity. College drift is the process by which parts of one college become incorporated into another. Although there is a degree of constant mobility within the socio-cognitive structure, especially in fragmented adhocracies, whole clusters of significant documents sometimes change their home college. College differentiation describes the process by which a broadly defined college splits up into several new colleges with a higher degree of specialization. It is thus a pattern of divergent evolution. College fusion occurs when two or more previously independent colleges merge into a single college.

### Co-citation analysis of the mindfulness field: Its intellectual structure

Our main objective for applying co-citation analysis was to provide the structure of the knowledge base of mindfulness literature—that is, the past of mindfulness—by determining the most influential works in the field.

#### Co-citation data and analysis

To identify the sample of primary documents, a keyword search for “mindfulness” was conducted in the Web of Science Core Collection database—the most frequently used database for this purpose (Zupic and Cater, 2015). The search imposed only one additional criterion—that the documents be published in English—which yielded 18,342 primary documents. Some of the main research areas featuring mindfulness literature were psychology (8,550 documents), psychiatry (4,002), neuroscience (1,534), education (1,032), public environmental health (790), and nursing (703). A closer look at the documents' publication dates revealed that the field recently has achieved exponential growth. Whereas, only 140 articles were published before 2000, more than 3,000 new publications were released in 2020 alone. The journal *Mindfulness* has published the most research in the field by far (with 1,216 documents), followed by *Frontiers in Psychology* (371), *Annals of Behavioral Medicine* (246), *Personality and Individual Differences* (198) and *PLOS One* (166). Eric L. Garland (102 publications), Javier Garcia Campayo (86), and Nirbhay N. Singh (72) are the most published authors in the field. The USA (8,513), the UK (1,819), and Australia (1,374) are the most represented countries (in terms of the first author's affiliation location). The University of Toronto (253), Harvard University (240), Harvard Medical School (230), and the University of Washington (230) are the most represented institutions.

Due to the high number of secondary documents that the search produced, a cutoff point, or citation threshold, was applied, which refers to the minimum number of citations by primary articles that a cited reference must have to be included in the analysis. Our main goals of employing a cutoff point were to select only the most influential documents and to limit the document set to a manageable size (Zupic and Cater, 2015). The general rule of thumb that is frequently applied in bibliometric visualizations (Caputo et al., 2021; Premru et al., 2022) is that one tries to plot the ca. 100–400 most important units; this provides optimal complexity while still being interpretable. For the co-citation analysis of the mindfulness literature, the 3,000 most relevant publications according to the Web of Science database were identified, and they were connected to 93,052 secondary articles. The citation threshold was set at a minimum of 33 citations, leaving 410 articles in the sample. The bibliographic data was imported into the software VOSviewer (Van Eck and Waltman, 2010).

#### Co-citation results

**Figure 1:**
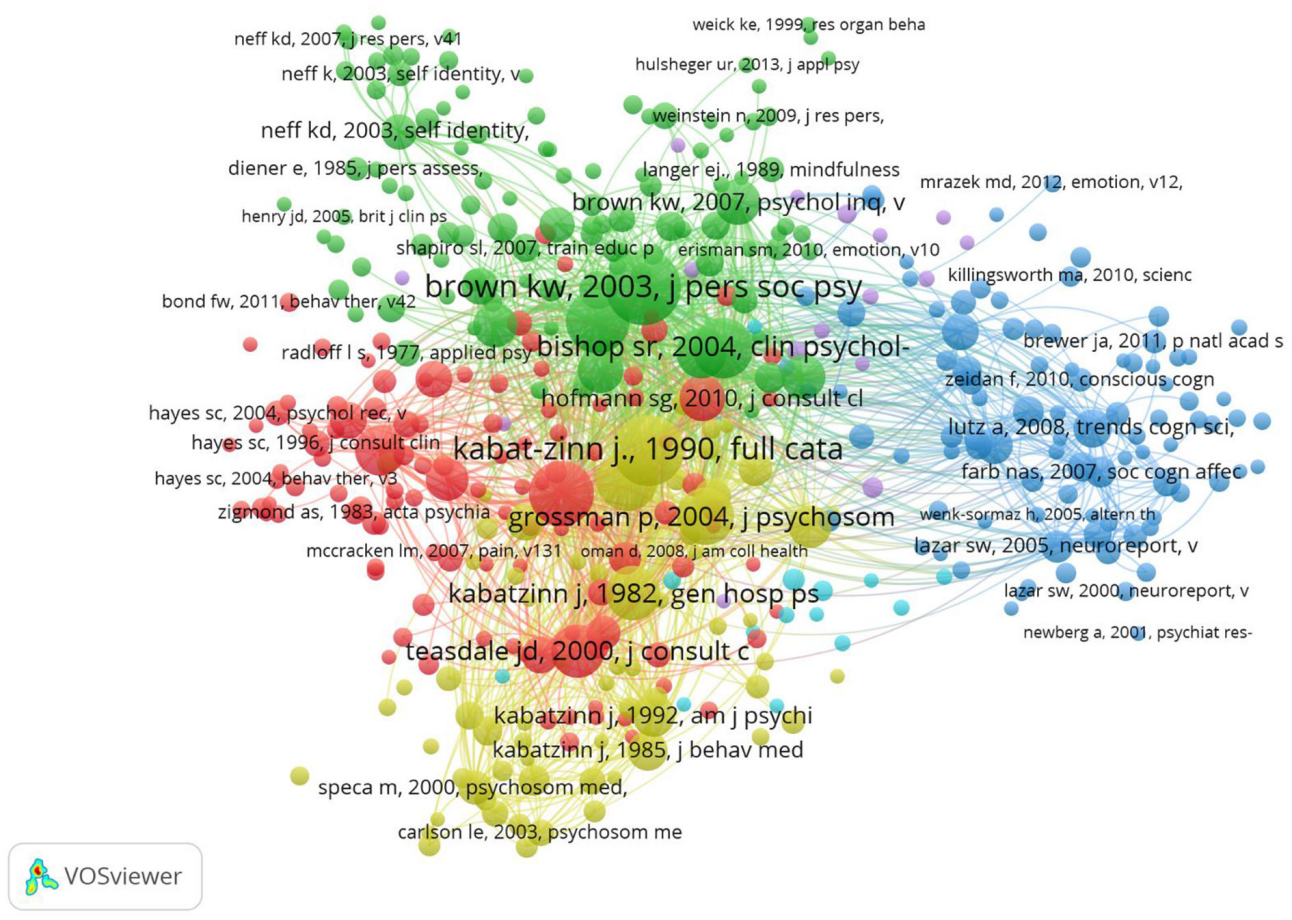
Network visualization of co-citation analysis.

VOSviewer allocated each of the 410 articles in the analysis to one of six clusters. An illustration of the network is shown in Figure 1. In illustrations pertaining to co-citation and bibliographic coupling results, each node represents one document (only first authors are named), and edges represent co-citations of two documents. Thicker edges indicate more co-citations. Node size indicates the number of other documents along which this node is co-cited (i.e., its degree), and darker color indicates greater strength (i.e., the sum of the weights of all edges to which a node is connected).

As Figure 1 shows, the research field is relatively densely arranged, except for the dark blue cluster, which seems to separate itself a bit from the rest of the research. This indicates a low level of fragmentation and strong cohesion between the subfields and authors of the respective articles of all other clusters. Furthermore, a fair amount of overlap exists between the green, red, yellow and purple clusters, where the clusters' borders were not as clear. Only the light blue and purple clusters were somewhat scattered throughout the network. Below, we describe each cluster in more detail.

##### Green cluster: Definition, measurement, and assessment of mindfulness

The first co-citation cluster, composed of 108 articles, was the most influential by far, as it contained three of the top five most cited articles in mindfulness research: Brown and Ryan (2003) with 13,806 citations, Bishop et al. (2004) with 11,203 citations and Baer et al. (2006) with 10,996 citations. The number of citations is likely so high because those articles documented the development of crucial measurement scales, among which are the MAAS (Brown and Ryan, 2003; Shapiro et al., 2007), the FFMQ (Baer et al., 2006), and the KIMS (Baer et al., 2004). An examination of the various questionnaires confirmed that authors have yet to agree on one definition of mindfulness, clarify it, or understand its operation (Brown et al., 2007). The KIMS identifies observing, describing, acting with awareness, and accepting without judgment as the four mindfulness skills (Baer et al., 2004), whereas the FFMQ also includes non-reacting as a facet of the construct (Baer et al., 2006). Bishop et al. (2004) and Brown et al. (2007) were motivated by this lack of clarity to propose a clear and consistent operational definition of mindfulness. The main challenge of the topic was to develop theoretically and empirically grounded models that link various aspects of mindfulness—that is, expression of mindfulness, processes underlying mindfulness effects, trait and state mindfulness relevant outcomes, and mindfulness interventions (Brown et al., 2007; Shapiro et al., 2007). We concluded that the articles in the green cluster constitute a foundational background for understanding the concept of mindfulness and its relationship to other variables (Baer et al., 2006; Fredrickson et al., 2008).

##### Yellow cluster: The mindfulness-based stress reduction program

The yellow cluster contained a total of 67 articles. Here, probably the most groundbreaking emerging work in the field of medicine and psychology refers to Kabat-Zinn's (1982, 1990) Mindfulness-Based Stress Reduction (MBSR) program. As a pioneer in the mindfulness field, he aimed to transfer the Buddhist mindfulness practice into Western culture and apply it as a complementary treatment for chronic pain patients through self-regulation processes (Kabat-Zinn, 1982). The results of his study showed a drastic reduction in perceived pain and mood disturbance. The MBSR program's ultimate goal has remained the same since its introduction: to reduce suffering and increase health and wellbeing. Because the program was not intended for the treatment of a specific condition, it has been applied to treat a wide range of conditions. For instance, it is used to reduce stress and mood disturbances, increase positive states of mind, and improve overall quality of life in cancer outpatients (Speca et al., 2000; Baer, 2003; Carlson et al., 2003; Grossman et al., 2004).

##### Red cluster: Mindfulness-based interventions

Analysis of the third cluster (112 articles) revealed which mindfulness-based interventions (MBIs) exist and which conditions they are used to treat. Two decades after the introduction of MBSR, Teasdale et al. (2000) introduced mindfulness-based cognitive therapy (MBCT) based on a combination of MBSR and cognitive behavioral therapy (CBT) strategies. This therapy was aimed at reducing relapse and recurrence in depressive patients. To assess MBCT's efficiency, Segal et al. (2002) proposed the Mindfulness-Based Cognitive Therapy Adherence Scale. Another popular intervention, developed by Hayes et al. (2006), is acceptance and commitment therapy (ACT), which is commonly used to treat post-traumatic stress disorder (PTSD) and substance abuse. Empirical research on mindfulness-based interventions has made a significant contribution to mindfulness research because such studies provide the instruments for the practical application of mindfulness. Moreover, mindfulness meditation significantly reduces anxiety and depression symptoms (Teasdale et al., 2000; Hofmann et al., 2010). The general consensus is that these interventions are beneficial and that their effects are rather robust and strong (Hofmann et al., 2010).

##### Dark blue cluster: Mindfulness mechanisms

The dark blue co-citation cluster, which contains 93 articles and is spatially more distant from the others, indicating a potential disconnection from an otherwise closely connected network of clusters, relates to the examination of underlying processes between mindfulness practice and its effects. The works of Jha et al. (2007), Lutz et al. (2008), and Hoelzel et al. (2011) represent the main publications of this cluster. These researchers tried to explain how mindfulness and MBIs really work using neuroimaging techniques such as functional magnetic resonance imaging (fMRI) to examine the effects of meditation on brain activity. Hoelzel et al. (2011) explored four mediators that could explain mindfulness outcomes: attention regulation, emotion regulation (ER), body awareness and change in perspective on the self. Lutz et al. (2008) and Chambers et al. (2009) explored how mindfulness meditation exerts its effects through ER. Another stream of research that evolved in the dark blue cluster related to linking mindfulness training and brain activity (Farb et al., 2007; Lutz et al., 2008; Hoelzel et al., 2011). The researchers aimed to explain how mindfulness interventions work by using neuroimaging to examine the effects of meditation on brain activity.

##### Purple cluster: Mindfulness for children and adolescents

A small and still fragmented cluster with 16 articles formed around the emerging field of mindfulness for children and adolescents. The research area is still in its infancy and requires more high-quality research and well-designed methodologies (Greenberg and Harris, 2012). Preliminary studies showed that school-based mindfulness programs that applied mindfulness and yoga interventions seem to have a positive impact on problematic responses to stress, such as rumination or emotional arousal (Mendelson et al., 2010). In addition, they help helping to build resilience (Greenberg and Harris, 2012) and assist with treating psychological disorders (Biegel et al., 2009). Similarly, Zylowska et al. (2008) showed that mindfulness training improved self-reported attention deficit hyperactivity disorder (ADHD) symptoms and performance on tasks measuring attention in adolescents.

##### Light blue cluster: Mindfulness and substance abuse

The light blue cluster is very small (14 articles) and relatively fragmented, and it mainly shows mindfulness practice as a treatment for substance use disorders (Witkiewitz et al., 2005; Bowen et al., 2006, 2009). Initial results indicate that the use of substances such as alcohol, marijuana, or crack cocaine is reduced after mindfulness-based treatment (Bowen et al., 2006).

### The field's development over time: Applying temporally-delineated co-citation analysis against the backdrop of the invisible colleges framework

**Figure 2:**
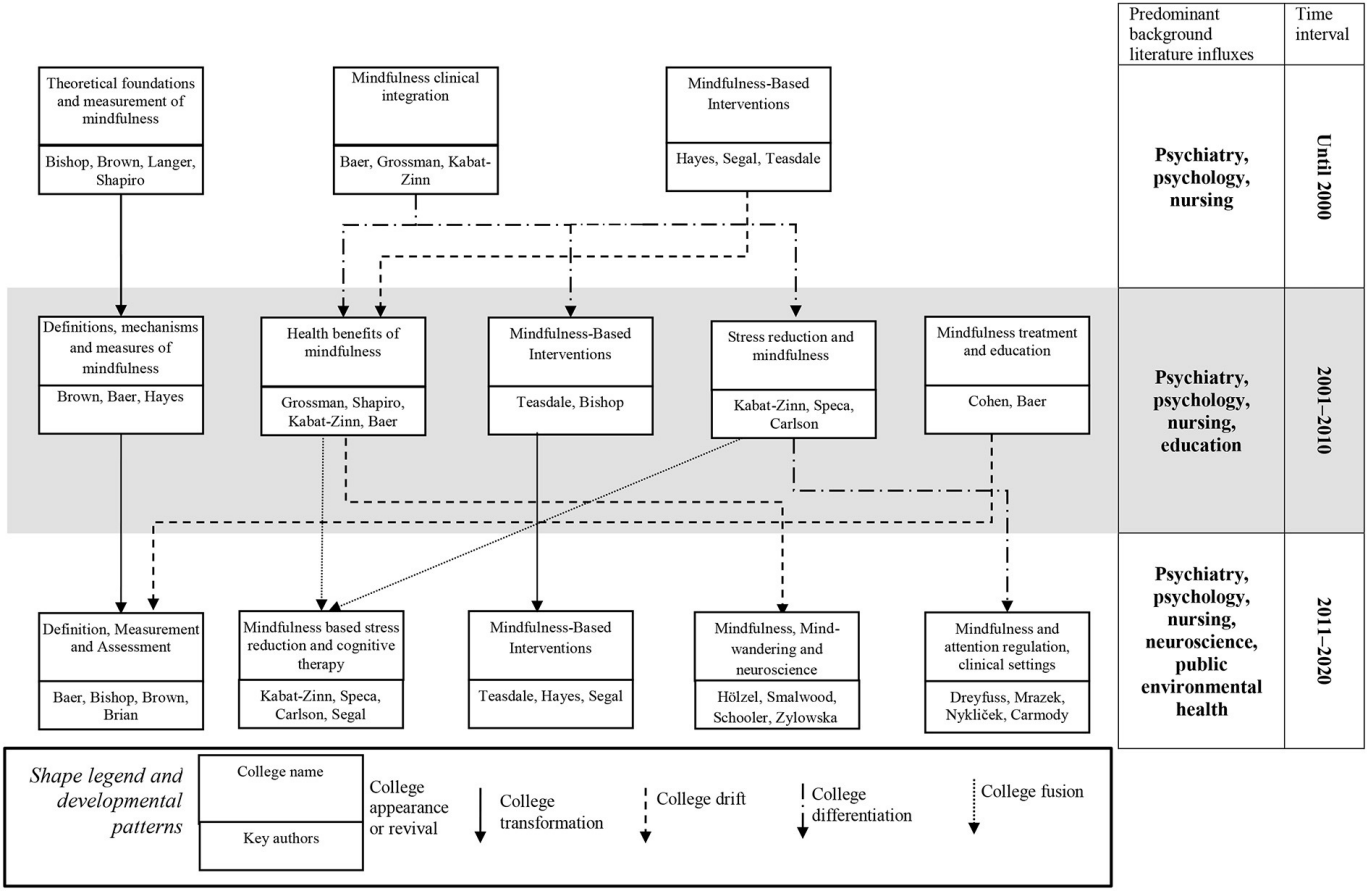
Development patterns of mindfulness research based on the invisible colleges framework.

To examine the field's development across time, we also conducted three co-citation analyses for the following time periods: up to 2000, 2001–2010, and 2011–2020 (see Appendix 1). We summarized the clusters identified this way in Figure 2, which is all-inclusive and stems from the application of the “invisible colleges” framework to the temporally-delineated co-citation results. The invisible colleges correspond to the clusters that were identified by the co-citation analysis. The invisible colleges are theories/backgrounds from the co-citation analyses across the time periods, but in the process of summarizing the invisible colleges, the clusters were condensed (merged) thematically further into a figure that would be more parsimonious and clearer.

Our results (see Figure 2) indicate that mindfulness research began in the following three invisible colleges: (1) theoretical foundations and measurement of mindfulness; (2) mindfulness clinical integration; and (3) mindfulness-based interventions. Key theoretical foundations in the first time period can be found in disciplines of psychiatry, psychology, and nursing. The first cluster can be considered a predominant research stream that has transformed into further clusters that retain the original focus, with some influxes from mindfulness treatment and education (which emerged during the second time period) in the final time period.

The second initial cluster (mindfulness clinical integration) differentiated into three separate ones during the second examined period: (a) health benefits of mindfulness, (b) MBIs, and (c) stress-reduction and mindfulness. The health benefits of mindfulness college fused with part of the stress reduction and mindfulness college to constitute mindfulness-based stress reduction and cognitive therapy in the final time period. The MBIs college has transformed further in a straightforward manner and retaining its original focus, whereas part of the health benefits of mindfulness college drifted into mindfulness, mind-wandering, and neuroscience. Part of the stress reduction and mindfulness college also differentiated into mindfulness and attention regulation, with a particular focus on clinical settings. In the final time period, disciplines of neuroscience and public environmental health also became important in providing theoretical bases for new influxes into the field.

### Co-word analysis: Key themes in mindfulness research

This technique was aimed at identifying key themes in the mindfulness field. The various clusters in the bibliographic map corresponded with the subfields of the mindfulness research area (Van Raan, 2014).

#### Co-word data and analysis

For co-word analysis, we used the same data set of 3,000 primary documents as for document co-citation. Because of the high number of primary documents, a cutoff point was applied to limit the number of keywords in the semantic map. Finding the right threshold (i.e., the frequency of keywords that end up making the final figure) in the co-word analysis required some trial and error. Initially, the idea was to set the threshold at a minimum of three citations, but that produced eight clusters, which would have been rather difficult to interpret. Finally, a cutoff point of 45 frequencies was chosen, such that out of 7,833 keywords, 112 different keywords met the threshold.

#### Co-word results

**Figure 3:**
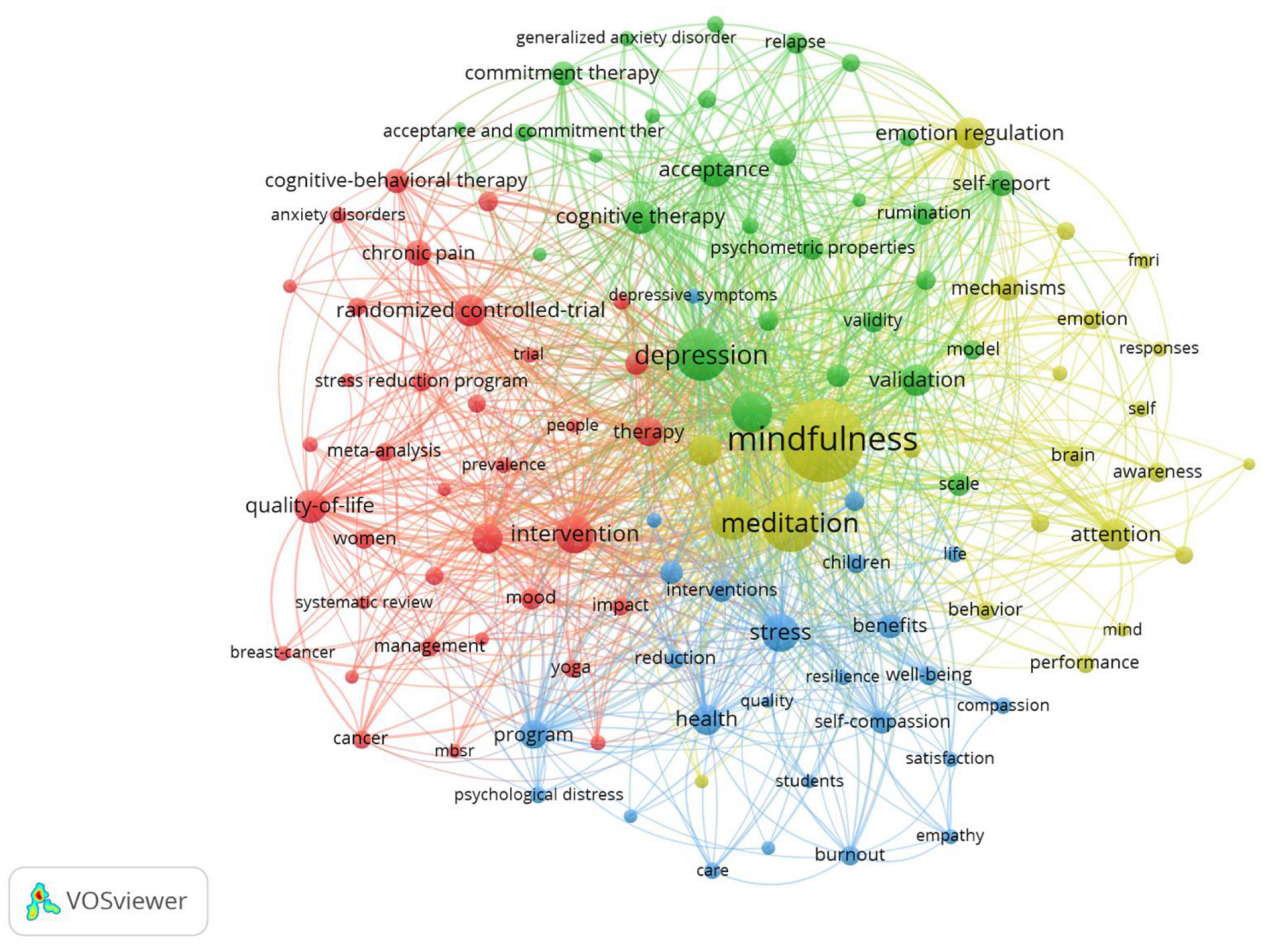
Network visualization of co-word analysis.

In this section, we present the results of the co-word analysis, which produced four clusters. As in the document co-citation analysis, each of the 112 keywords that met the minimum threshold of 45 citations was allocated to one of the clusters by VOSviewer. As shown in network illustration (Figure 3), the clusters are relatively clearly separated from each other. The two most connected keywords, and thus those displayed with the biggest circles, were “mindfulness” and “meditation.” Although allocated to the yellow cluster, they are actually central to the whole research field and cannot really be observed as pertaining to only one cluster. Meditation is central because it represents the main approach with which mindfulness is practiced in all the mentioned interventions.

In the next section, we examine every cluster in further detail.

##### Blue cluster: Benefits of mindfulness

The blue cluster, which is composed of 25 keywords, laid out some of the positive effects of engaging in mindfulness, which can increase wellbeing significantly (Chiesa and Serretti, 2009). This can be achieved by reducing symptoms of a physical illness or mental disorder in a clinical population or by directly reducing stress in a non-clinical population. “Satisfaction,” “happiness,” “self-compassion,” “resilience,” and “mental health” were other keywords that appeared in the cluster, representing the benefits mindfulness can provide for an individual. It is self-explanatory that increased physical and mental wellbeing lead to improved satisfaction and happiness. Self-compassion results from mindfulness through heightened awareness and acceptance and is complemented with kindness toward oneself aimed at easing suffering (Neff, 2003). Interestingly, the cluster also contained the keywords “adolescents,” “children,” and “students,” which could be considered a small subcluster of the blue cluster. This demonstrated the relevance of recent studies that investigated if and how mindfulness practices could be used in schools (e.g., Diamond and Lee, 2011).

##### Red cluster: Physical health conditions

The red cluster (containing 35 words) indicated how MBIs can effectively improve physical health conditions. This content relates, for example, to the Stress Reduction and Relaxation Program, which later became the MBSR program, took shape. Kabat-Zinn's (1982) research, for example demonstrated that mindfulness has significant power in decreasing perceived suffering. In many cases, the pain reduction amounted to more than 50% (Kabat-Zinn, 1982). In addition, mindfulness can also be beneficial for patients undergoing cancer treatment (Speca et al., 2000; Carlson et al., 2003). In addition to the keywords “cancer,” “breast cancer,” and “chronic pain,” the cluster also features terms such as “symptoms,” which could refer to any bodily symptom, and “sleep,” which might be connected to the fact that patients experiencing severe pain often report trouble sleeping. Thus, mindfulness can improve sleep quality (Carlson and Garland, 2005) and overall quality of life.

##### Green cluster: Mental disorders

Mental disorders might be the area to which MBIs are most commonly applied. In the green cluster (29 keywords), the network clustered the terms “depression,” “anxiety,” “generalized anxiety disorder,” “post-traumatic stress-disorder,” and “relapse prevention” connected to patients suffering from substance abuse. The empirical evidence that mindfulness produces improvements in these domains is very strong (Kabat-Zinn et al., 1992; Teasdale et al., 2000; Hofmann et al., 2010). The cluster also featured keywords that describe the behavior of an individual suffering from a certain disorder. “Rumination” is very common in patients with depression, as is “experiential avoidance,” even though such patterns create harm in the long term (Baer et al., 2004). “Acceptance and commitment therapy” and “mindfulness-based cognitive therapy,” other terms in this cluster, are well-established interventions used to treat the mental disorders mentioned above.

##### Yellow cluster: Mindfulness mechanisms

The yellow cluster is made up of 23 keywords and addresses the mechanisms of mindfulness. This is a topic of current investigation, and no clear answer has yet been found to the question “How does mindfulness work?” Shapiro et al. (2006) proposed that mindfulness arises when, in addition to intention, “attention” and “awareness,” two keywords in this cluster that are part of the larger concept of “consciousness,” are activated simultaneously. Another branch of research has looked into how mindfulness is connected to “emotion regulation” and “self-regulation,” other important terms in this cluster. Researchers have agreed that ER is a central part of mental health and that many forms of psychopathology are attributable to dysfunctional ER (Hayes and Feldman, 2004). If mindfulness heightens emotional awareness, it can help an individual take notice of their emotions earlier and regulate them more easily (Price and Hooven, 2018). Mindfulness also facilitates choosing appropriate responses to one's emotions (Brown and Ryan, 2003).

Another topic that appears in the content analysis is the measurement of mindfulness, but this is scattered a bit among the clusters. This essential part of any research field groups keywords such as “questionnaires,” “validity,” “reliability,” and “psychometric properties.” Several mindfulness questionnaires have been developed in the past (e.g., MAAS, KIMS, FFMQ), and fellow researchers have thoroughly tested them for validity and reliability, demonstrating that they have good psychometric properties (Baer et al., 2006).

### Bibliographic coupling: The current state of the art of mindfulness research

Bibliographic coupling analysis was applied to evaluate current trends in the mindfulness field, detect popular topics, and identify opportunities for future research. This interpretation is done in two ways: (1) using identified popular streams based on trends highlighted in recent years or (2) by pointing out opportunities for additional connections among subfields that are not sufficiently connected, but could or should be, based on their content (e.g., because of thematic or semantic similarity, complementarity or theoretical connectedness).

#### Bibliographic coupling data and analysis

**Figure 4:**
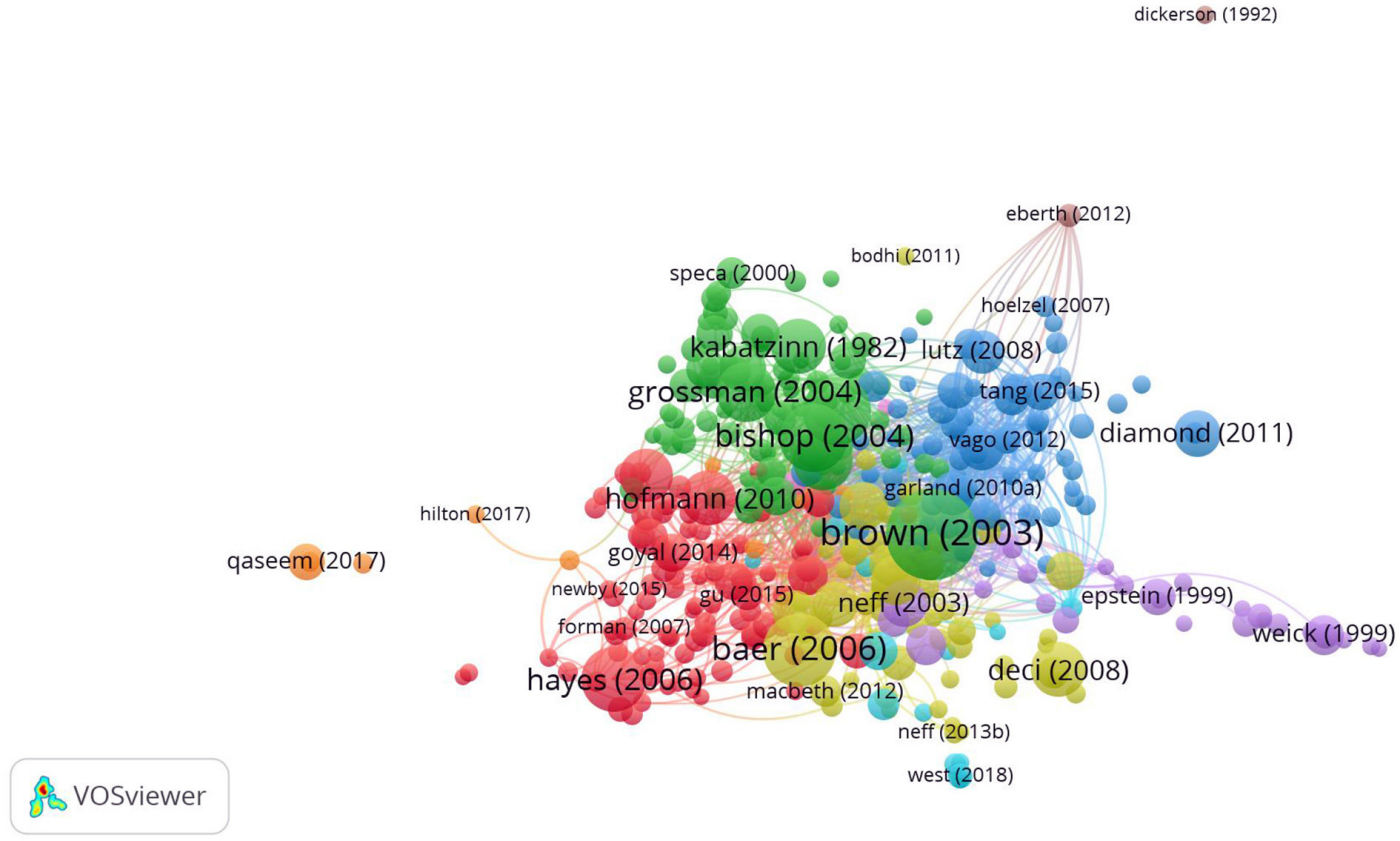
Network visualization of bibliographic coupling.

We applied the same data set used for document co-citation and co-word analysis in bibliographic coupling, this time to consider the future orientation of mindfulness research. The citation threshold was set at 160, and 363 out of 3,000 primary documents met this threshold. We chose this cutoff point to capture the most relevant publications and reduce the data set so it was small enough to be interpretable. The same procedure as described above was employed, yielding nine clusters, as shown in Figure 4.

#### Bibliographic coupling results

In the bibliographic coupling analysis, the cluster labels were not based exclusively on the clusters' biggest circles because a significant share of them were among the 25 most cited articles, which do not necessarily represent the research front, but rather its knowledge base. Instead, newer articles with smaller nodes were also considered, which helped us derive the cluster labels. This section presents the results of the five biggest clusters (i.e., most impactful). The brown and pink clusters—the latter of which is not visible in Figure 4, as it is located under the green cluster—were treated as outliers because they contained only two documents each, and these exhibited no similarity in terms of content. Also, the orange and light blue cluster were not taken into account in this analysis due to the small number of articles in those clusters. Interestingly, in the network illustration, all the articles were clustered extremely densely together, especially in the center of the illustration. However, to the right and left sides, the clusters included a few articles at the periphery of the network. Two documents, Diamond and Lee (2011) and Qaseem et al. (2017), had a high citation count–755 and 1,219, respectively—but very low link strengths of 68 and 59, respectively. This indicated a relatively weak relation regarding the similarity in content between these articles and the rest of the cluster or even the bibliographic coupling network.

##### Red cluster: New directions in cognitive behavioral therapy and emotion regulation

Until the middle of the twentieth century, psychological disorders were managed mainly with medication and physical treatments. However, with the rise of behavior therapy (BT) in the 1950s, the focus shifted to eliminating non-adaptive behavior and promoting adaptive behavior (Rachman, 2015). Cognitive therapy (CT) emerged in the 1960s because BT did not show the desired effects in the treatment of depression. CT deals with maladaptive cognitions and, as a remedy, instead of trying to eliminate the maladaptive cognitions completely, introduces an approach aimed at correcting and adapting them (Rachman, 2015). Finally, the two approaches merged into CBT in the 1980s, which is now recommended for the treatment of anxiety disorders, PTSD, and obsessive-compulsive disorder (OCD) (Rachman, 2009). In recent years, a third generation of BT has evolved, which consists of contextual approaches from BT and CT (Hayes et al., 2011). According to Hayes et al. (2006), the central difference between CBT and these newer approaches is that CBT promotes changing psychological events, whereas contextual approaches are aimed at changing the function of these events and an individual's relationship with them. The main strategies for achieving this objective are acceptance, mindfulness, and cognitive defusion (Masuda et al., 2004). Among the interventions that are part of the third generation, MBCT shows effectiveness in preventing depressive relapse and recurrence (Teasdale et al., 2000; Ma and Teasdale, 2004; Piet and Hougaard, 2011). Zautra et al. (2008) drew a direct comparison between CBT and mindfulness meditation in treating arthritis patients with depression and found that patients with recurrent depression benefited most from the mindfulness meditation intervention. Furthermore, the cluster included publications in which MBIs were applied to treat anxiety disorders (Roemer and Orsillo, 2007; Vøllestad et al., 2012), chronic pain (McCracken and Vowles, 2014; Veehof et al., 2016), and substance use disorders (Chiesa and Serretti, 2014).

Finally, we identified a small emotion regulation subcluster within the red cluster. Hayes and Feldman (2004), Feldman et al. (2007), Chambers et al. (2009), and Webb et al. (2012) investigated how mindfulness and ER are connected. In general, ER is referred to as the adaptation of one or more elements of emotional experience or response (Chambers et al., 2009). Chambers et al. (2009) showed that the majority of psychological disorders have deficient ER at their core. Mindfulness has used to successfully treat a range of psychological disorders, and it seems to promote adaptive ER. Both mindfulness and ER, are relatively new fields of psychological investigation, and consensus is still lacking on clear operational definitions (Chambers et al., 2009). The red cluster, which was made up of 90 articles in total, consisted mainly of explorations of MBIs and acceptance-based interventions that form part of the new generation in CT and BT, as well as a small subcluster of ER literature.

##### Green cluster: Beneficial effects of mindfulness-based interventions

The green cluster (84 articles) comprised some of the most foundational works in the mindfulness field. Many of the articles in this cluster were published around 2004 or earlier. Influential studies included definitions of mindfulness (Kabat-Zinn, 1982; Bishop et al., 2004), reviews of MBIs' effectiveness (Baer, 2003; Grossman et al., 2004; Hofmann et al., 2010), and the development of the MAAS (Brown and Ryan, 2003). The beneficial effects of mindfulness include improved psychological wellbeing, self-regulation, more positive emotional states, reduced mood disturbance and stress, reduced negative affect, improved coping and vitality, and improved awareness of the moment-to-moment experiences of mental processes and external events (cf. Garland et al., 2015). These results have been confirmed by several meta-analyses, controlled trials, and empirical studies (cf. Speca et al., 2000; Carmody and Baer, 2008).

##### Yellow cluster: Positive psychology and mindfulness

The yellow cluster, composed of 62 articles, mainly comprised research in two subclusters of positive psychology: self-compassion and self-determination theory. The foundational article of Neff's (2003) pioneering self-compassion theory was assigned to the purple cluster by VOSviewer, when it actually fit perfectly into this yellow cluster. According to Neff, self-compassion builds on three components: self-kindness, common humanity, and mindfulness. Furthermore, Neff and Beretvas (2013) examined the role of self-compassion in romantic relationships, and Pace et al. (2009) drew a connection between compassion meditation and stress reduction responses. Fredrickson et al. (2008) found that loving-kindness meditation can increase personal resources, such as purpose in life or social support, when mediated by positive emotions. Deci and Ryan's (2008) article on self-determination theory—a theory characterized by motivation, personality development, and wellness—was also included in the yellow cluster. However, it was located at the periphery of the bibliographic coupling network; thus, it was not closely connected to the mindfulness construct.

##### Dark blue cluster: The neuroscience of mindfulness and mindfulness for children and adolescents

Most research in this cluster (70 articles) examined how the practice of mindfulness can evoke changes in brain structure and brain functioning (Tang et al., 2007, 2015; Chiesa et al., 2011; Hoelzel et al., 2011; Vago and Silbersweig, 2012). Moore and Malinowski (2009) found that experience in mindfulness meditation correlates positively with improvements in attentional functions and cognitive flexibility. These effects on attention can have a long-term impact on the brain (Lutz et al., 2008). Many of the studies mentioned above were supported by neuroimaging instruments used to detect which brain regions activated during meditation and whether there was a difference between meditating and non-meditating samples. The neuroscientific study of mindfulness is still in its infancy, and further research is needed to support the existing findings (Tang et al., 2015). Lutz et al. (2008) proposed that the field could benefit from a longitudinal study further exploring the long-term neurological effects of mindfulness meditation on brain functioning and structure.

The dark blue cluster also featured a small subcluster in which several scientific papers related to mindfulness for children and adolescents were clustered together. The central research included studies on MBIs in schools (Zenner et al., 2014) and meditation training for adolescents with ADHD (Zylowska et al., 2008). Moreover, Diamond and Lee (2011) found that in children aged 4–12 years, mindfulness can foster executive functions (creativity, flexibility, self-control, and discipline), which are important aspects of cognitive control and critical contributors to children's performance. According to Zoogman et al. (2015), overall, MBIs were beneficial for children and adolescents. However, these studies mostly investigated healthy youths; thus, future research could investigate the efficacy of interventions for children and adolescents in clinical settings.

##### Purple cluster: Mindfulness at work

In the last cluster, composed of 26 articles, a majority of the documents were centered on the topic of mindfulness in the workplace. Good et al. (2016), whose study was originally assigned to the orange cluster, but fits in perfectly with the purple cluster in terms of content and is surrounded by purple nodes in the network, identified how the influence of mindfulness on attention affects emotion, cognition, behavior, and physiology. These, in turn, can have a positive effect on workplace outcomes such as performance, wellbeing, and work relationships. Glomb et al. (2011) suggested that improved workplace performance and wellbeing stem from the self-regulatory characteristics of mindfulness. By being more attentive and aware of their thoughts, emotions, and behaviors, employees can adjust better particular situations. The leading theory regarding task performance is Dane's (2011) contingency theory, which is based on the assumption that wide attentional breadth induced through mindfulness practice is positively related to task performance. When employees reduce mind-wandering during work hours with the help of mindfulness, their task performance is likely to increase due to their heightened focus on the task at hand. The field of research in mindfulness at work is still young, but the beneficial effects of mindfulness appear to spill over into the workplace environment.

More recently, research has begun exploring the benefits of team and collective mindfulness. Sutcliffe et al. (2016) defined collective mindfulness as “the collective capability to discern discriminatory detail about emerging issues and to act swiftly in response to these details” (p. 56). They also presented evidence implying that collective mindfulness is linked not only to lower turnover rates on the employee level, but also to higher customer satisfaction, greater innovation, more effective allocation of resources, and improved quality, safety, and reliability on the organizational level. Particularly strong effects of this kind have been found in complex and dynamic work environments.

## General discussion

Mindfulness is gaining considerable interest not only from researchers, but also from many employers and individuals. This paper helps position mindfulness within the broader clinical psychology and psychiatry field and provides an objective and comprehensive overview of the existing mindfulness literature. The use of three bibliometric techniques—document co-citation, co-word analysis, and bibliographic coupling—allowed us to tap into the past to reveal the underlying intellectual structure of the field and its development over time, into the present to identify the central mindfulness topics, and into the future to identify relevant directions for future research.

### Theoretical contributions

The document co-citation analysis shows that the articles with the most international impact form a strong network with high cohesion and relatively low fragmentation. Nevertheless, the various subfields in the co-citation network can be clearly identified. Studies that developed mindfulness assessment and measurement scales constitute an important component of the intellectual structure, as they are among the articles cited most often. The FFMQ provides the most comprehensive coverage of the various aspects of mindfulness in the general population (Bergomi et al., 2013). In line with previous bibliometric reviews (e.g., Wang et al., 2021; Daniel et al., 2022), our bibliometric coupling results point out the importance of the development of the MAAS (Brown and Ryan, 2003), probably one of the most widely used mindfulness assessment scales in the research to date. Moreover, the document co-citation analysis yielded a subcluster with a range of MBIs and therapies that incorporate mindfulness and studies that examine the efficacy of these interventions. Researchers were able to demonstrate that the application of mindfulness led to positive health outcomes in many different contexts. Notably, our bibliometric review showed that MBSR achieved a breakthrough among mindfulness interventions (Grossman et al., 2004). The research confirmed MBSR's effectiveness as an intervention for the treatment of psychological and physical symptoms (see Baer, 2003; Bishop et al., 2004). The co-citation analysis also revealed that the investigation of the mechanisms of mindfulness forms part of the knowledge base. However, although these mechanisms have been explored since the early days of mindfulness research, this topic still poses many questions and requires further study (Tang et al., 2015). Unlike previous bibliometric reviews we indicate above, our co-citation analysis revealed a promising and emerging field of mindfulness for children and adolescents, and substance use. Perhaps this is not surprising as mindfulness interventions are reinforced to be introduced in schools (Zenner et al., 2014) and proven its efficacy in substance use disorders treatment (Vinci et al., 2021).

In addition to conducting a static co-citation analysis, we also explored the evolution of the mindfulness field, mapping its trajectory and development by applying the invisible colleges framework (Vogel, 2012). This enabled us to identify and describe clusters of colleges of the field's intellectual foundations (i.e., studies researchers in the field have cited during specific periods) and track how the predominant perspectives have changed over time. Document co-citation is indeed a dynamic measure that evolves as older documents accumulate more citations (Batistič et al., 2017). Thus, co-citation frequencies might shape a certain intellectual field and may be helpful when detecting shifts in certain schools of thought (Pasadeos et al., 1998). We portrayed evolutionary patterns of dynamic change in the field, showing the developmental path of specific schools of thought and how they have changed over time. This provides interested researchers with a more nuanced understanding of how various sub-sections of the field have developed over time and where theoretical influxes into specific streams originated at a specific point in time.

In the co-word analysis, the clusters overlap more within the network than in co-citation. However, there is also overlap between the co-citation and the co-word networks in that both feature the cluster “mindfulness mechanisms.” Thus, in comparison to previous co-word analyses (i.e., Baminiwatta and Solangaarachchi, 2021; Wang et al., 2021), we found that understanding the mechanisms of mindfulness plays a significant role in advancing the mindfulness research overall.

In addition, the co-word network yielded a cluster that encompasses the beneficial effects of mindfulness, which range from stress reduction to improved psychological wellbeing, satisfaction, happiness, resilience, self-compassion, and mental health. Furthermore, similar to Baminiwatta and Solangaarachchi (2021) and Wang et al. (2021), the co-word analysis shows that mindfulness has been applied in relation to treating mental health problems such as depression and anxiety. In addition, our co-word analysis showed mindfulness training to be also highlighted as a potentially successful treatment for physical health conditions, such as cancer and chronic pain.

The documents in the bibliographic coupling network cluster together relatively densely, except for a couple of peripheral articles on one side of the network. This indicates that the research front is relatively well-organized and that scientists seem to work in the same direction to advance the field as a whole. In the first cluster, the evolution from BT to CBT to newer mindfulness- and acceptance-based approaches was presented. Other clusters that the bibliographic coupling revealed were “beneficial effects of MBIs,” which includes many of the foundational works in the field, and “positive psychology and mindfulness,” where a connection between the two domains was made through the topics of self-compassion and self-determination theory.

The analysis also revealed interesting and relatively under-researched areas such as mindfulness for children and adolescents. For example, a recent meta-analysis confirmed the beneficial effects of mindfulness for non-clinical and clinical youth (Zoogman et al., 2015), and there is emerging research on the development of mindfulness scales for children and the introduction of MBIs in school settings (Kuby et al., 2015). Similarly, mindfulness continues to contribute to clinical practice and medical research (Montero-Marin et al., 2015; Qaseem et al., 2017; West et al., 2018). Similar to Daniel et al. (2022) bibliometric coupling results that exclusively linked domains of business management to mindfulness research, our bibliometric review also revealed that the dominant domain of the research frontier of the field highlights benefits of practicing mindfulness at work (Glomb et al., 2011; Good et al., 2016) and the benefits of the evolving concept of collective mindfulness (Sutcliffe et al., 2016).

In addition to consolidating these detailed insights into mindfulness research in one place, this paper contributes to the field by complementing extant qualitative reviews and meta-analyses. Analyzing such a large sample of articles allowed us to provide a broader and more objective overview than would have been possible with other forms of literature review. This bibliometric review also offers a strong methodological backbone, a property that a number of mindfulness studies lack. Other relevant contributions are the network illustrations, which offer the reader a graphical description of the research field, facilitate understanding, and make comparisons easy (Zupic and Cater, 2015). Finally, the combination of the three bibliometric techniques facilitated deeper insights into the complex multidisciplinary field of mindfulness.

### Future research recommendations

One of the central strengths of this bibliometric approach is its ability to uncover potential avenues for future research. This section points out various areas of the mindfulness literature that exhibit opportunities to deepen and clarify the current findings and apply mindfulness to entirely new contexts.

#### Refining the mindfulness concept

Before anything else, however, the mindfulness concept must be refined. Researchers call for methodologically sound studies and randomized clinical trials with larger sample sizes so that previous studies can be replicated and the beneficial effects of mindfulness confirmed (Baer, 2003; Davidson et al., 2003; Burke, 2010). Essentially, more evidence that is empirically sound must be collected to create a firm research base. Brown et al. (2007) also pointed out that one of the biggest challenges is developing good theoretical models that examine “the directional links between those conditions that support the unfolding and expression of mindfulness (e.g., attitudes like acceptance), mindfulness itself, processes explaining its effects (e.g., insight), and relevant outcomes of mindful states, traits, and interventions” (p. 231). Furthermore, Baer (2003) and Carmody and Baer (2008) suggested that to distill the effects of mindfulness, an MBI (e.g., MBSR) could be compared to a stress reduction program. Such a study would allow researchers to determine whether the effects identified are unique to MBSR or if the stress reduction program also leads to increased mindfulness.

#### Mindfulness at work and collective mindfulness

In the last decade, researchers have begun investigating whether mindfulness can also be beneficial in workplace settings. Current research includes explorations of the effects of mindfulness on task performance (Dane, 2011), job performance, turnover intentions (Dane and Brummel, 2013), workplace relationships (Glomb et al., 2011; Good et al., 2016), working memory capacity, mind-wandering (Mrazek et al., 2013), work engagement (Gunasekara and Zheng, 2019), and job satisfaction (Hüelsheger et al., 2012). Because many employers have taken interest in the topic of mindfulness at work, the field could benefit from further large, well-designed studies that verify these preliminary findings. Another field worthy of a closer empirical look is that of collective mindfulness, which could be investigated on the team and organizational levels.

#### Mindfulness and relationships

Another potential avenue for future research is mindfulness in relationships. This could include various kinds of relationships, including workplace relationships (Good et al., 2016), romantic relationships (Neff and Beretvas, 2013), familial relationships, and friendships. Carson et al. (2004) introduced the Mindfulness-Based Relationship Enhancement program, which was adapted from the MBSR. The results of their study indicate that the intervention increased relationship satisfaction, acceptance of one another, and closeness and reduced relationship distress (Carson et al., 2004). It would be interesting to explore whether mindfulness facilitates good communication. Overall, more research is needed in this domain to validate the existing research findings and to explore additional facets of mindfulness effects on relationships.

#### Mindfulness for children and adolescents

As described in the section on the yellow bibliographic coupling cluster, initial studies found that mindfulness can have beneficial effects on children and adolescents. However, because MBIs were applied only to healthy children and adolescents in these studies, future research should apply MBIs to young clinical populations to see if the effects can be replicated in this context. Another topic of potential investigation is determining whether MBIs need to be adapted (e.g., by introducing a playful aspect or gamification) to be more accessible to young children, who will likely find it difficult to sit still and focus on something as abstract as their minds during a meditation exercise.

#### Mindfulness in clinical practice

Mindfulness continues to contribute to clinical practice and is gaining more popularity in medical research. For example, research shows that non-invasive treatments such as yoga and CBT practices are effective treatments for chronic low back pain (Qaseem et al., 2017). However, researchers have called for more evidence that can provide clinical recommendations on the effectiveness of non-invasive low back pain treatments. Similarly, more research is needed on the effectiveness of mindfulness mediation for chronic pain overall (Hilton et al., 2017). Veehof et al. (2016) concluded that acceptance and commitment therapy (ACT) was more effective in treating depression in individuals with pain than mindfulness-based treatments were. Another emerging research front links mindfulness, physician wellbeing and the quality of patient care. However, longitudinal studies of burnout's effects and the impact of mindfulness interventions for physician burnout are needed (West et al., 2018). Finally, studies continue to confirm the beneficial effects of mindfulness on mental health and cognitive performance (Goyal et al., 2014; Tang et al., 2015). However, this stream of research is relatively new, and the underlying neural mechanisms and changes in the brain remain unclear.

### Limitations

Bibliographic methods have certain limitations, so they cannot stand alone and should be complemented by other review types, such as meta-analyses or narrative reviews. First, the error rate in citation databases is estimated at about 7–9%. If the data are not corrected and the errors not taken into account, the results of the bibliometric review might be distorted (Wallin, 2005). Moreover, citation patterns can vary widely among document types (Wallin, 2005). For example, the number of cited documents in a meta-analysis is many times higher than that of an article in an emerging field where not much literature exists yet. The results of a bibliometric analysis can also be distorted through frequent self-citation by some authors because this automatically increases the citation count (Zupic and Cater, 2015).

It is also impossible to establish the exact reason a certain publication was cited based solely on the bibliometric data. Those reasons can even differ among researchers who cited the same publication (Zupic and Cater, 2015). As Wallin (2005) also observed, although bibliometric methods are actually quantitative, they are sometimes used to make judgments about research quality. When a document is cited frequently, it should be regarded as an impactful publication with a high degree of international visibility rather than an article of good quality (Wallin, 2005). In addition, for newer publications, which naturally have fewer citations, it can be difficult to keep up with older, more frequently cited publications in bibliometric reviews, especially when applying the co-citation technique, as mentioned previously.

However, low citation rates could also simply indicate that a document pertains to a narrow field of study. Thus, they should not be misinterpreted as evidence of poor quality (Wallin, 2005). Once the bibliometric software produces the network output, the author of a bibliometric review must interpret the clusters, and they must be careful “not to try to fit the analysis to their existing preconceptions” (Zupic and Cater, 2015, p. 448). Otherwise, the analysis could lose its objectivity, which is a crucial advantage of bibliometric techniques. Bibliometric software (in our case, VOSviewer) does not provide the possibility for calculating indexes, as would be the case in standard network analysis, leaving the interpretation of the clusters to visual inspection. The size and position of specific documents in the clusters are included in specific figures, providing researchers with an idea of the most impactful studies.

In addition, the clusters the bibliometric software generates are not based on an exact science. This particularly goes for the choice of thresholds, which ultimately decide on the number of documents or keywords will be included on the figures. However, regardless of the threshold, the most important documents are included, and papers that are grouped together strongly also will be grouped in another analysis, which is the most important aspect of providing an accurate and comprehensive depiction of the research field in question.

The temporal analysis of the field's development over time is also not without limitations. Although the division of the observation period into three calendrical decades makes possible contrasting comparisons that reveal significant changes in the socio-cognitive organization of mindfulness research, other choices might have led to the detection of colleges that have remained invisible in this study. To gain a more fine-grained understanding of mindfulness research that displays a greater number of intermediate steps in the evolution of invisible colleges, a study's overall period could be divided into shorter contrasted intervals, provided that the accumulated citations are worth processing using bibliometric methods. Alternatively, future studies could switch from a comparative static design such as the one applied in this study to dynamic approaches to network analysis.

Future research could also explore whether topics and approaches taken by specific subsections of the field differ based on researchers' characteristics (e.g., gender, age, country, institutions) or on characteristics of the fields of journals that published this research, delving into specific disciplines and their predominant theoretical and empirical approaches. This would help researchers better understand the field, its development, and current trends.

## Data availability statement

The raw data supporting the conclusions of this article will be made available by the authors, without undue reservation.

## Author contributions

All authors listed have made a substantial, direct, and intellectual contribution to the work and approved it for publication.

## Conflict of interest

The authors declare that the research was conducted in the absence of any commercial or financial relationships that could be construed as a potential conflict of interest.

## Publisher's note

All claims expressed in this article are solely those of the authors and do not necessarily represent those of their affiliated organizations, or those of the publisher, the editors and the reviewers. Any product that may be evaluated in this article, or claim that may be made by its manufacturer, is not guaranteed or endorsed by the publisher.
