# Supplementary material for: Exploring the past, present, and future of the mindfulness field: A multitechnique bibliometric review
Source: Front Psychol. 2022 Jul 26;13:792599. doi: 10.3389/fpsyg.2022.792599 (PMC9361873; doi:10.3389/fpsyg.2022.792599)
Supplement: Supplementary file 1 [file Data_Sheet_1.docx]

Appendix 1.

CO-CITATION BY TIME PERIODS

**1. Up to 2000**

**
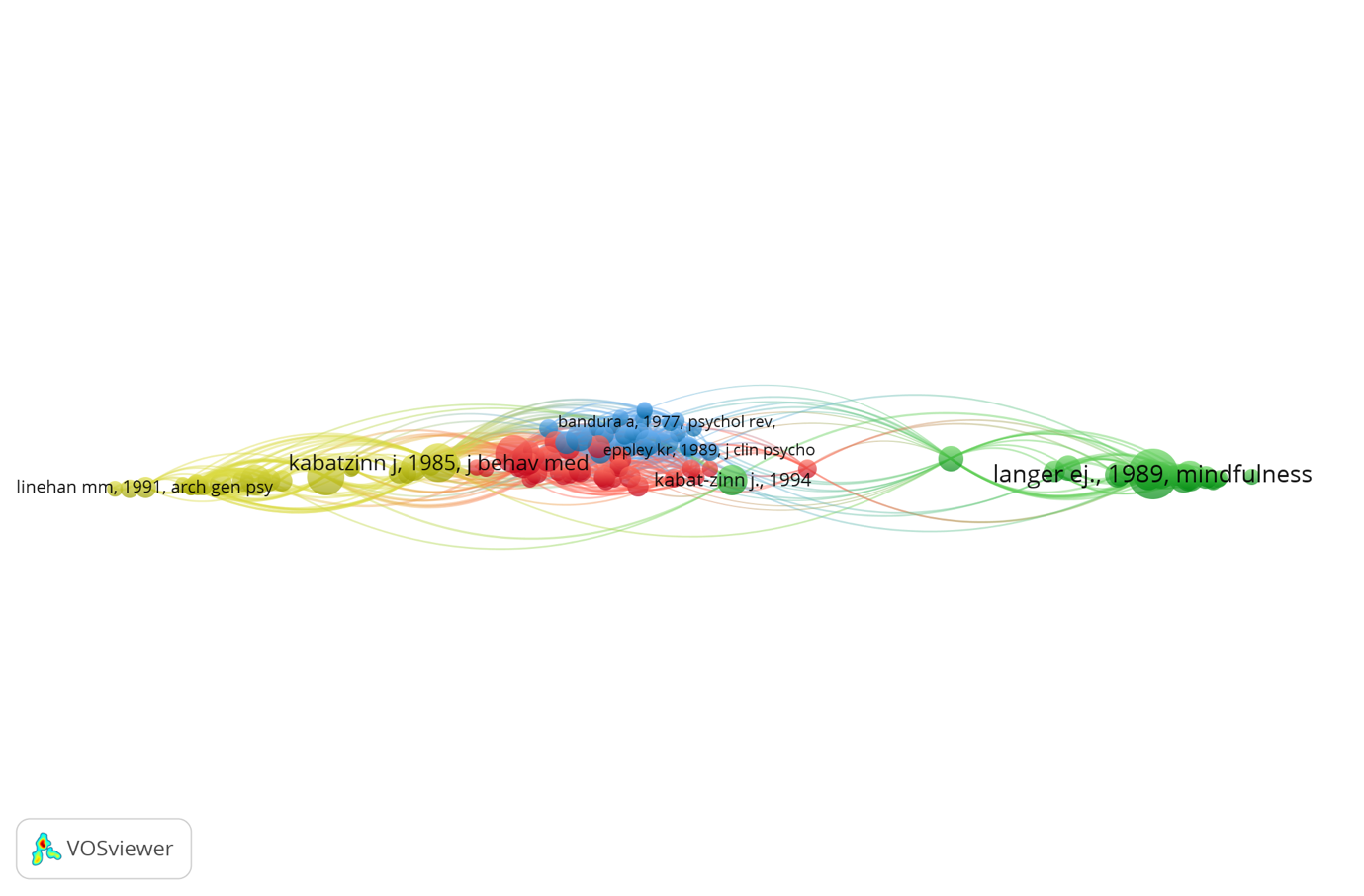
**

Up to 2000, threshold of 3, 124 articles


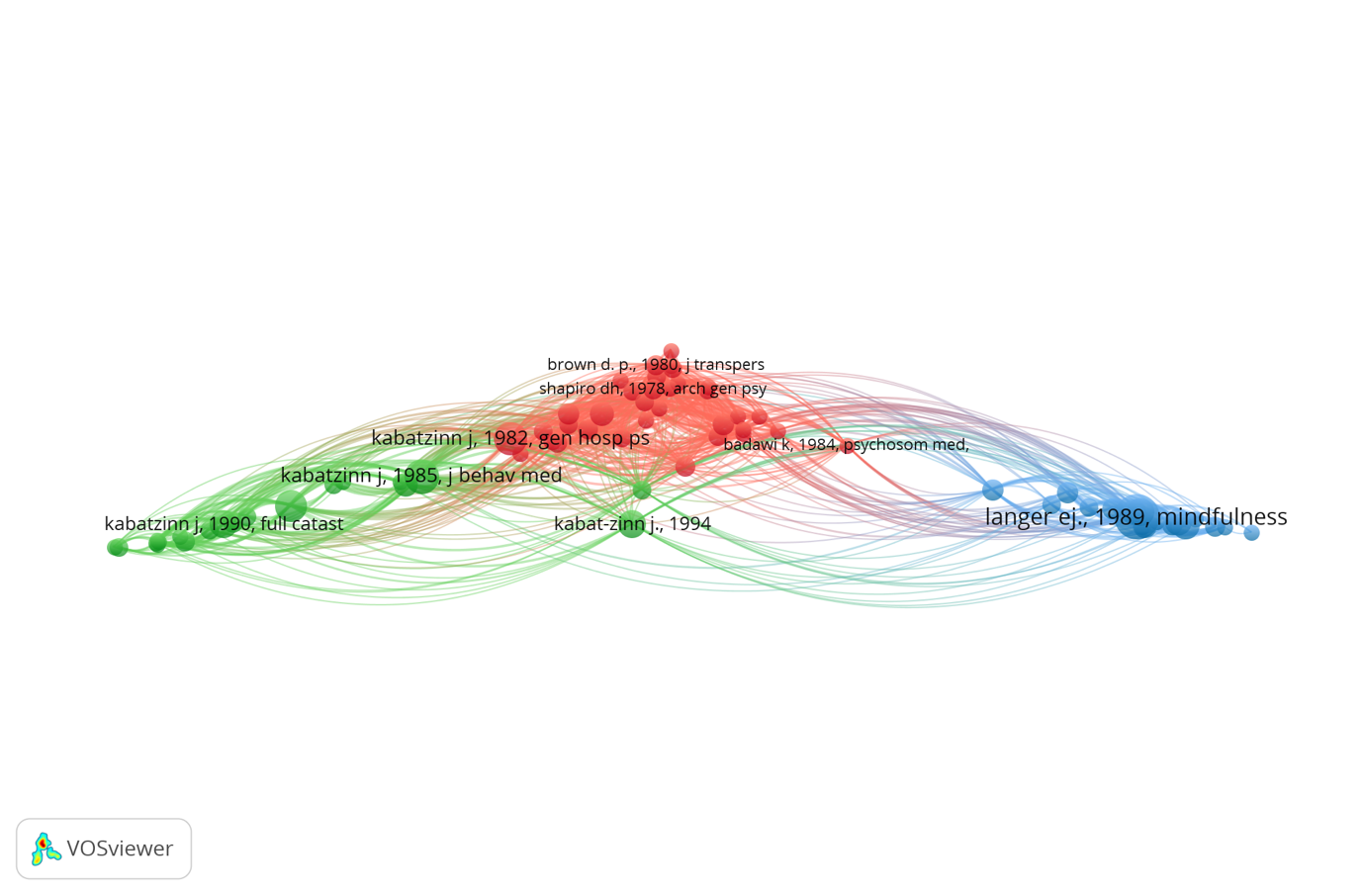


Up to 2000, threshold of 4, 73 articles


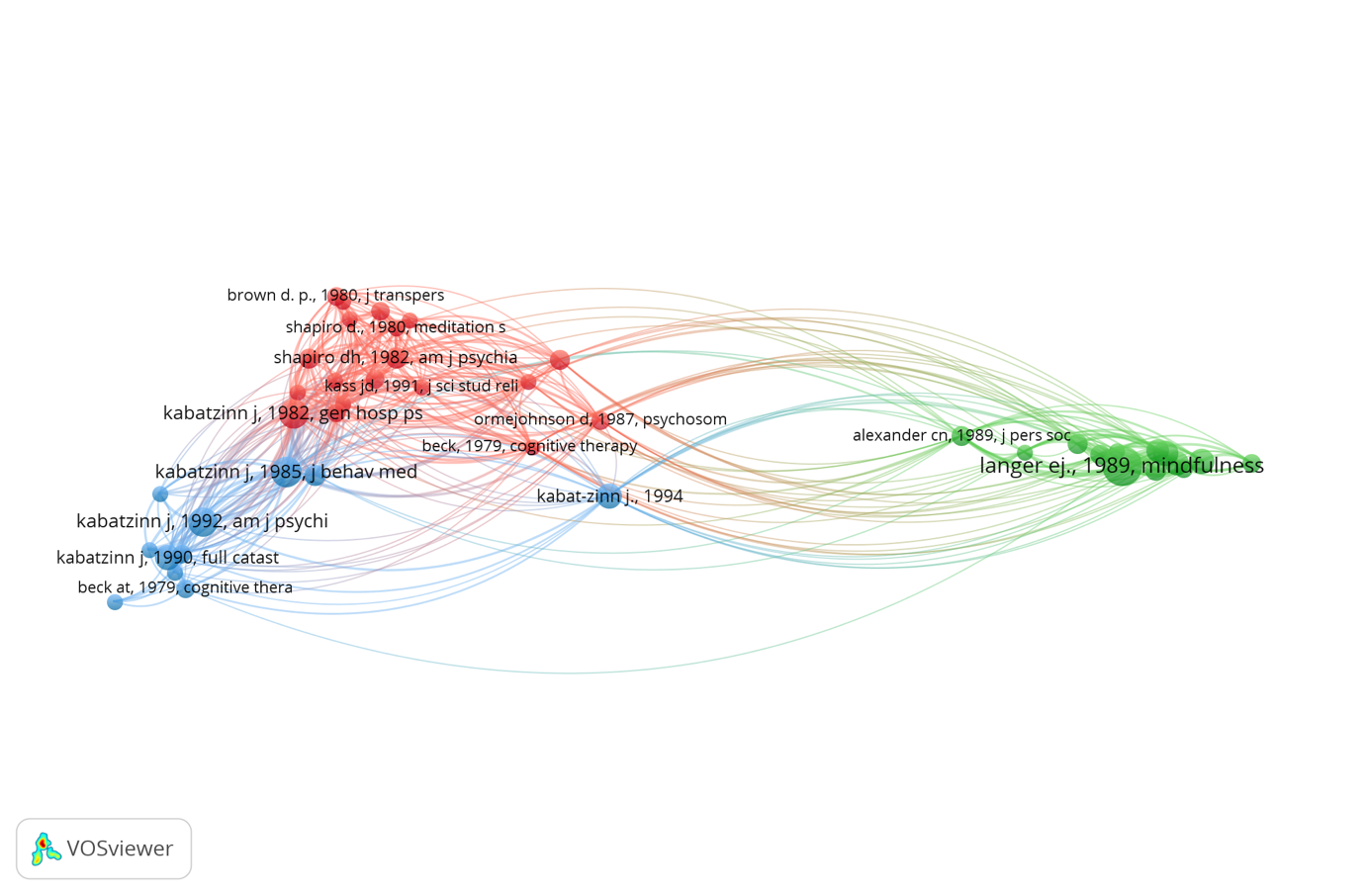


Up to 2000, threshold of 5, 43 articles

**2. 2001-2010**

**
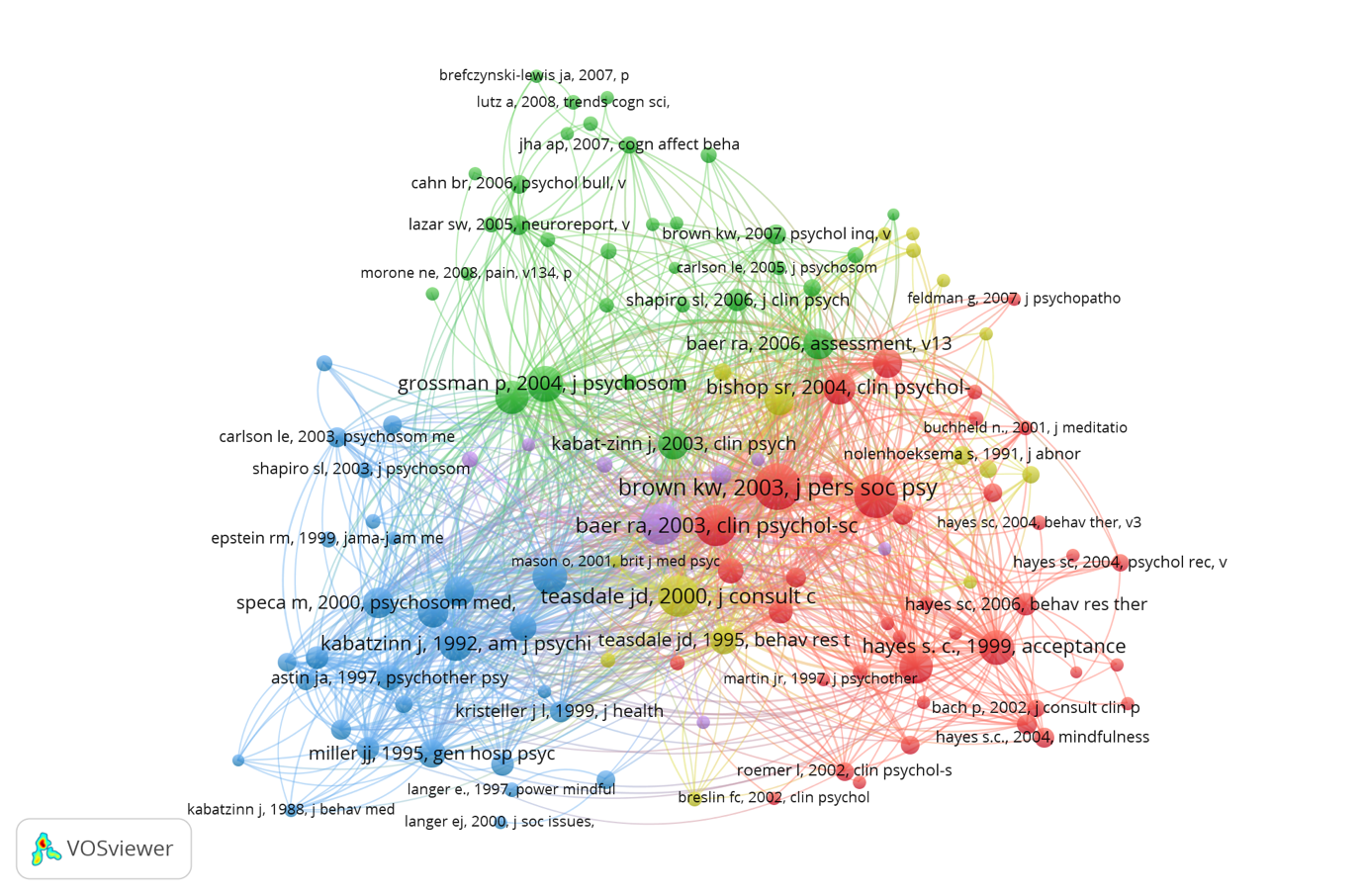
**

2001-2010, threshold of 25, 124 articles


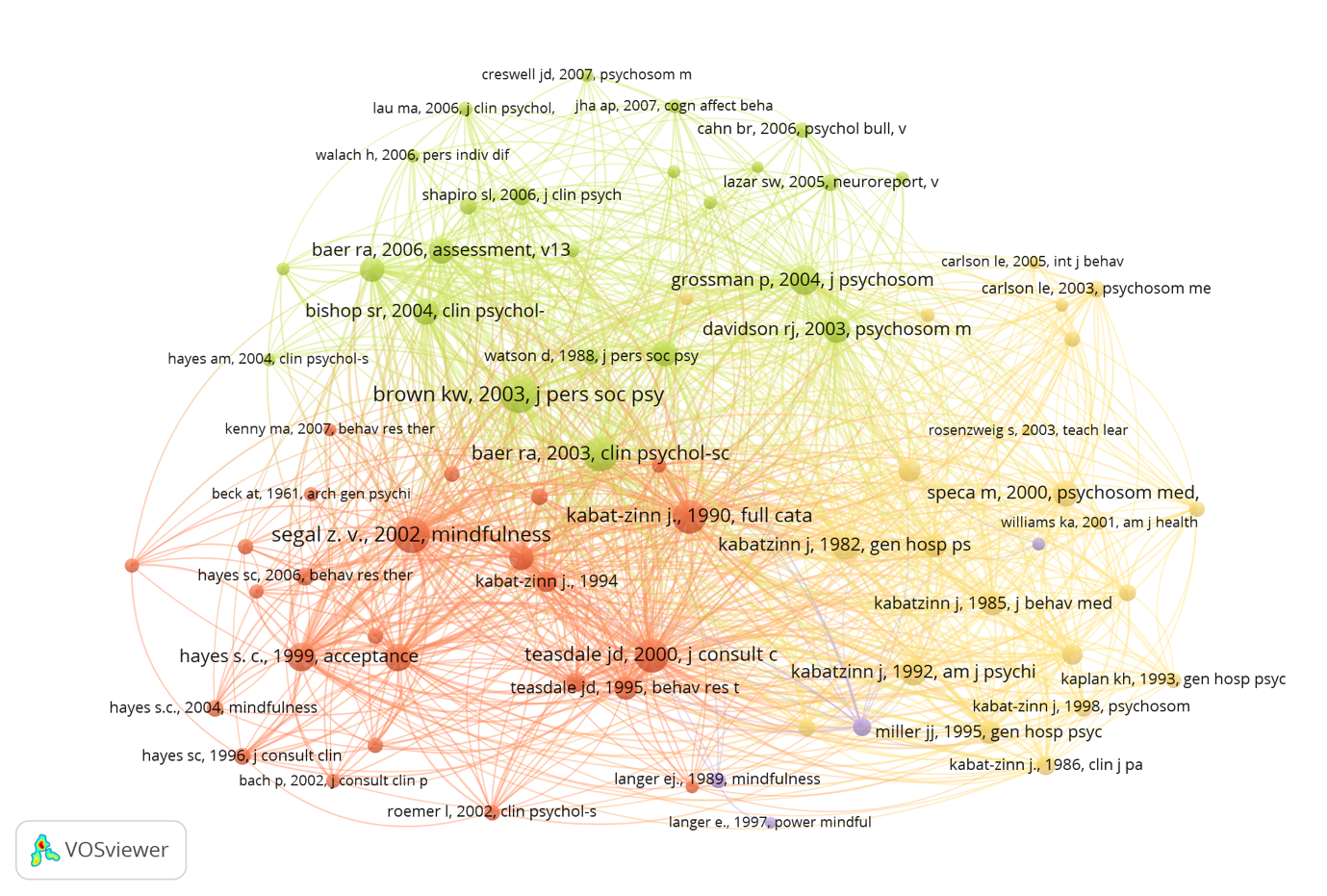


2001-2010, threshold of 35, 75 articles


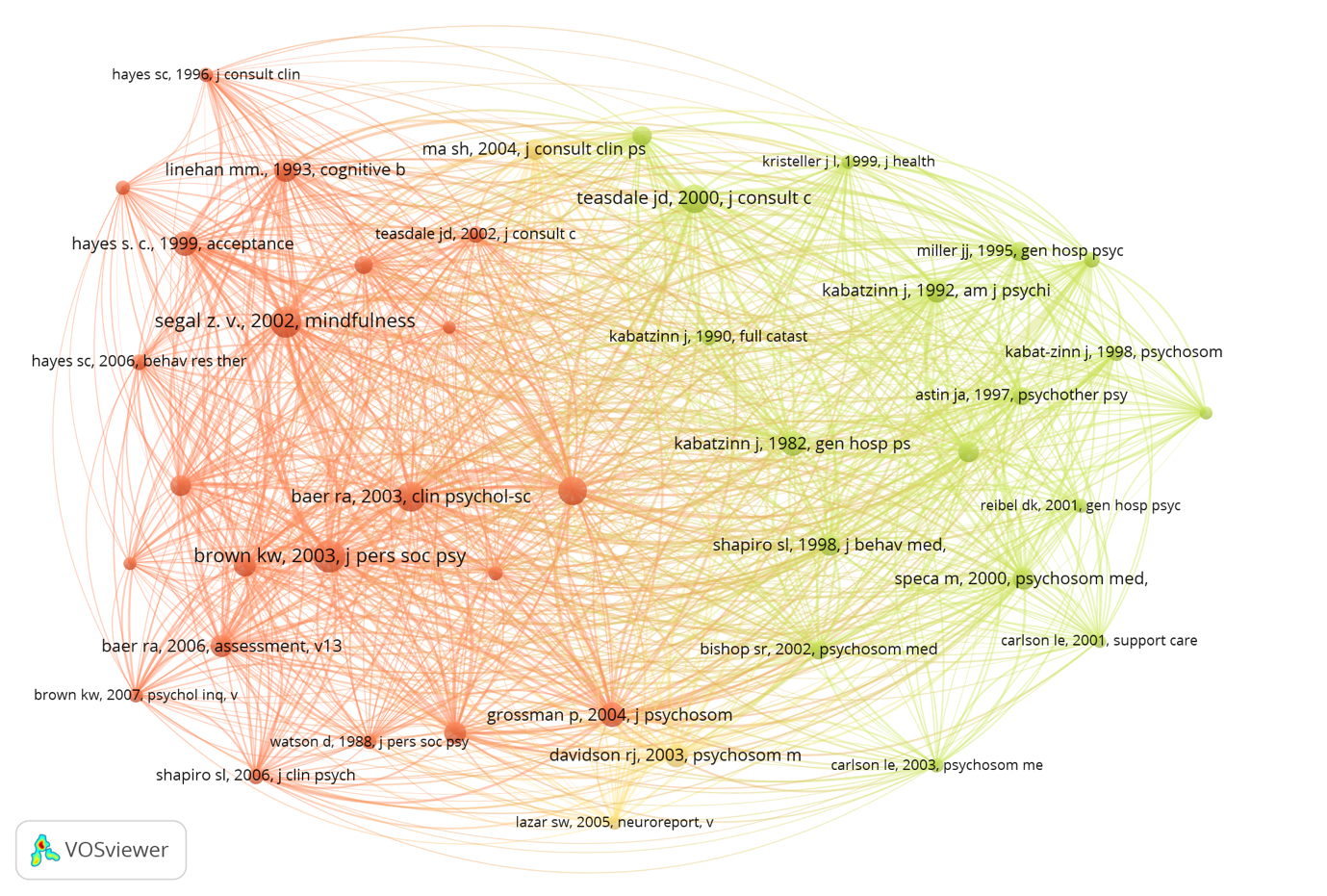


2001-2010, threshold of 55, 43 articles

**3. 2010-2020**


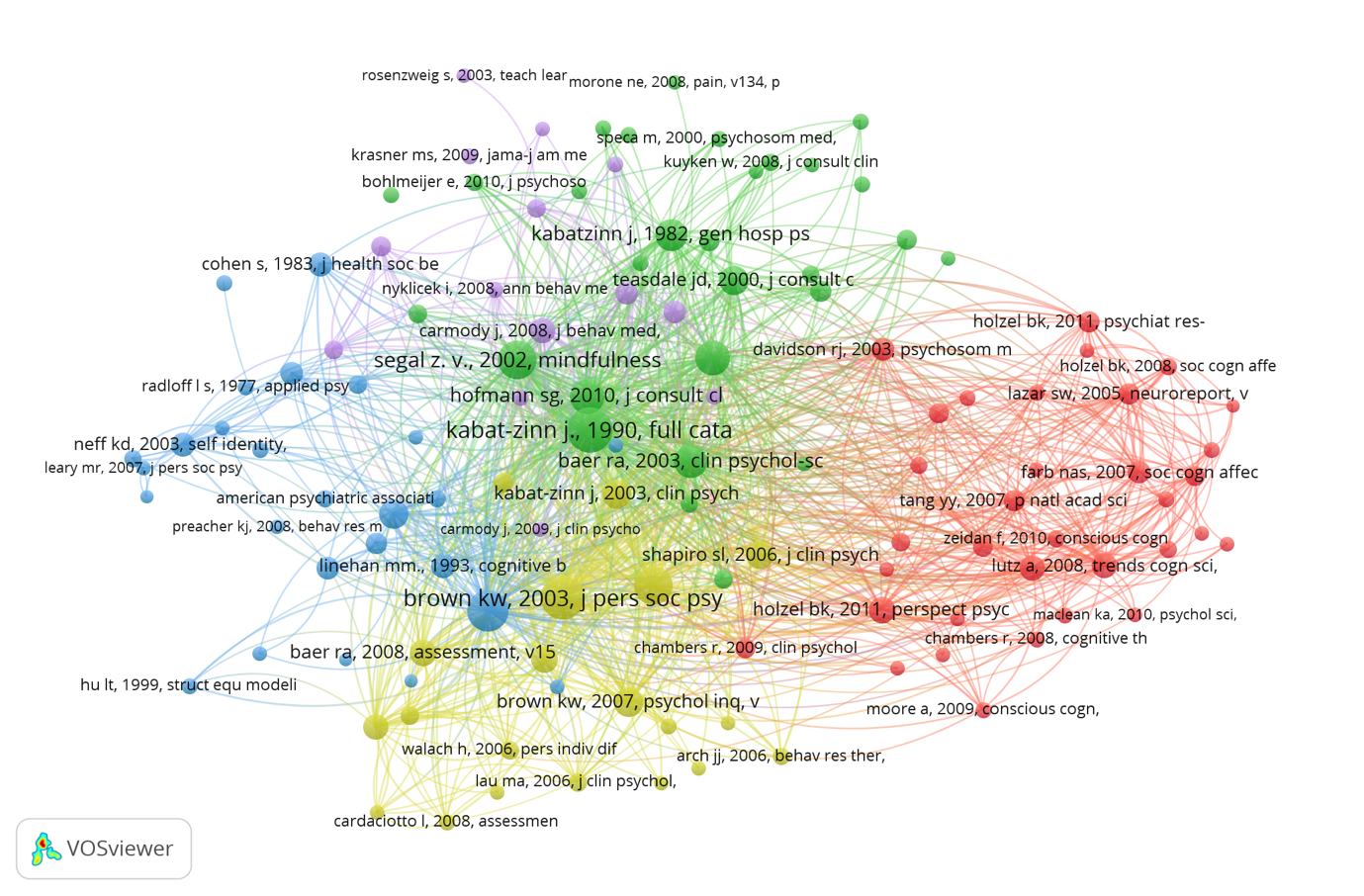


2010-2020, threshold of 45, 123 articles


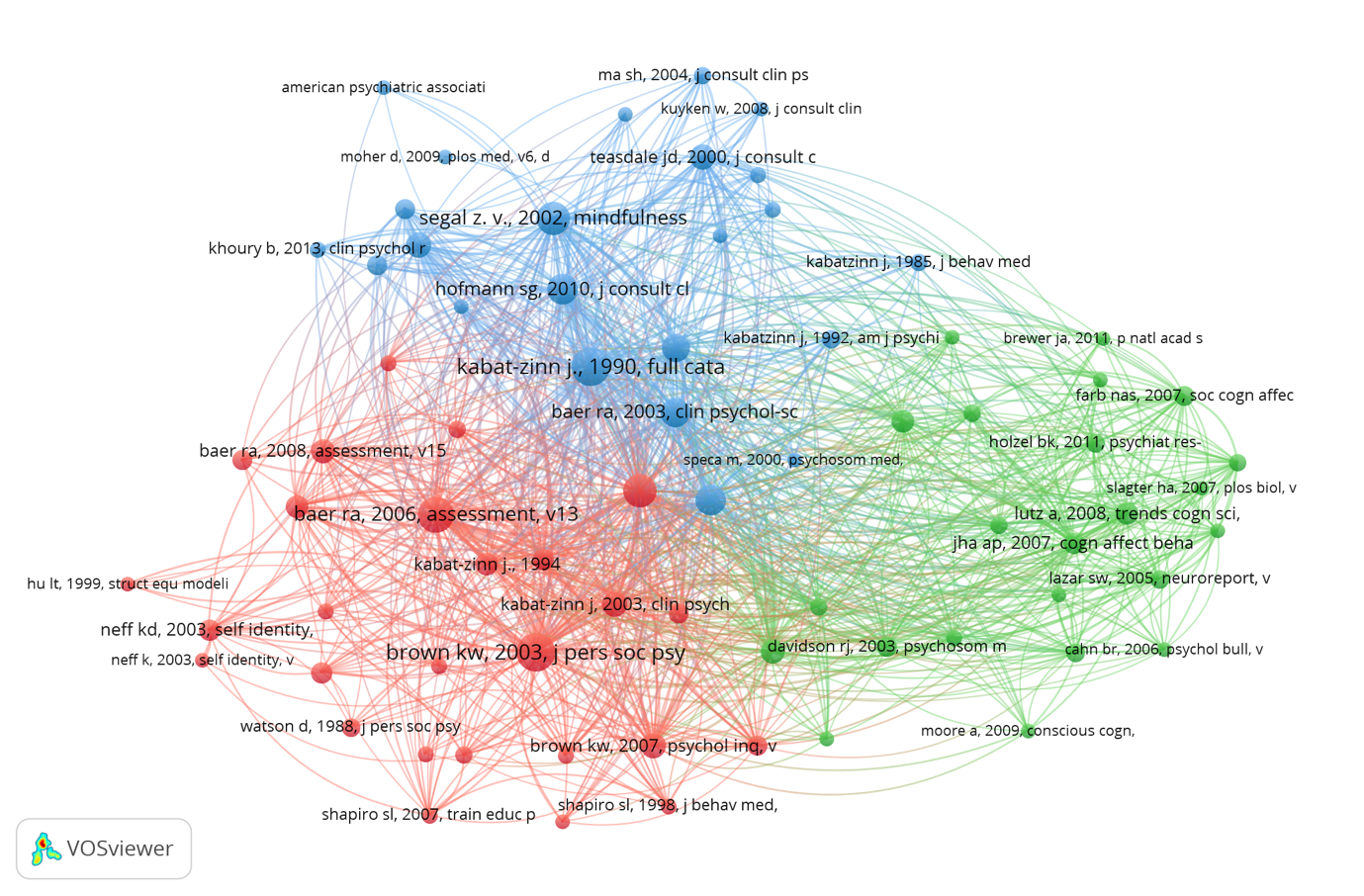


2010-2020, threshold of 61, 73 articles


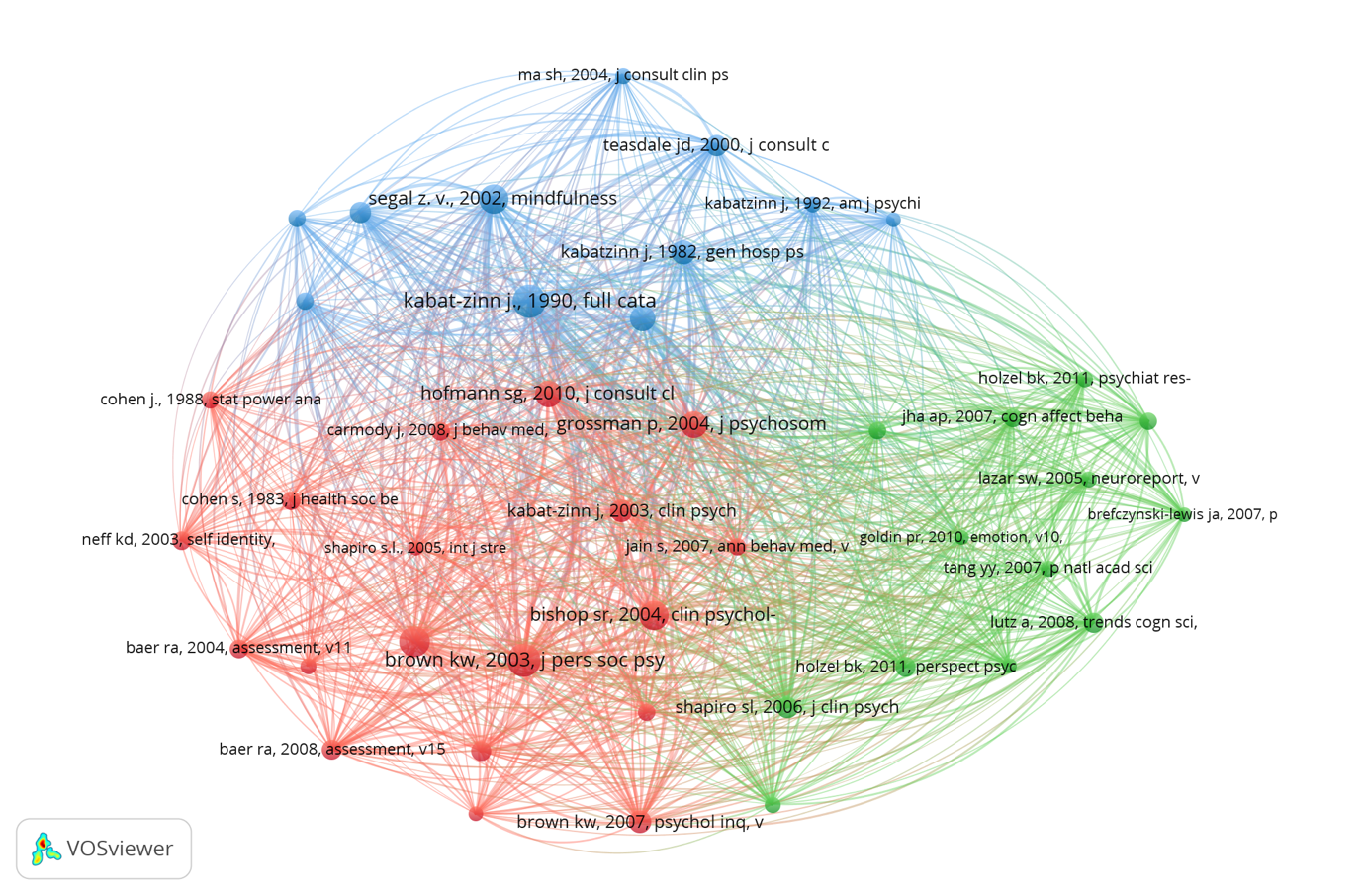


2010-2020, threshold of 82, 43 articles
